# Association of cumulative exposure and dynamic trajectories of the C-reactive protein-triglyceride-glucose and its modified indices with cardiovascular disease in individuals with cardiovascular-kidney-metabolic syndrome stages 0–3: a longitudinal analysis based on CHARLS

**DOI:** 10.1186/s12933-026-03197-x

**Published:** 2026-05-11

**Authors:** Xinyu Yin, Jingyuan Yang, Rongji Li, Meiyun Zhang, Huili Cao, Bin Yang

**Affiliations:** Department of Cardiology, The Second Hospital of Shanxi Medical University, #382 Wuyi Road, Xinghualing District, 030000 Shanxi Taiyuan, China

**Keywords:** Cardiovascular-kidney-metabolic syndrome, cardiovascular disease, C-reactive protein-triglyceride-glucose index-Chinese Visceral Adipose Index, CHARLS

## Abstract

**Background:**

To evaluate the incremental predictive value of modifying the C-reactive protein-triglyceride-glucose (CTI) index with obesity parameters for incident cardiovascular disease (CVD) across Cardiovascular-Kidney-Metabolic (CKM) syndrome stages.

**Methods:**

Based on the longitudinal CHARLS cohort, Cox proportional hazards was utilized. Predictive increments were assessed using time-dependent Nearest Neighbor Estimation (NNE) AUC with Bootstrap resampling, cNRI, and IDI. Pathophysiological mechanisms were explored via Weighted Quantile Sum (WQS) regression and causal mediation analyses.

**Results:**

After adjusting for demographic and clinical confounders, CTI and its modified indices all maintained robust, independent associations with incident CVD. Specifically, except for CTI-BMI, the new index, which incorporates the obesity index, transforms the dose-response relationship from a complex, nonlinear pattern to a threshold linear association. While increments in overall discrimination (AUC) were marginal, both baseline and cumulative CTI-CVAI significantly improved risk reclassification(cNRI: 0.093–0.126, IDI: 0.024–0.026, *P* < 0.001). The CKM stratification reveals stage-dependent predictive effects, with amplified relative risks in CKM 0–2 stages and stronger incremental value for risk reclassification in the CKM 3 stage. WQS confirmed visceral adiposity as the dominant risk driver, and physical frailty was identified as a significant pathophysiological mediator of the observed associations.

**Conclusions:**

Incorporating obesity indicators, particularly CVAI, into the CTI framework significantly improves the reclassification of CVD risk in early to moderate CKM stages. However, the lack of significant discriminative (AUC) improvement necessitates a careful clinical trade-off between adopting complex composite metrics and maintaining screening feasibility.

**Trial registration:**

Not applicable.

**Supplementary Information:**

The online version contains supplementary material available at 10.1186/s12933-026-03197-x.

## Introduction

Epidemiological data surveys indicate that the total number of global cardiovascular disease(CVD) cases nearly doubled from 271 million in 1990 to 598 million in 2025 over the 35-year period, making it a leading cause of the global disease burden [1–3]. Multiple studies have revealed a complex and close association between CVDs, chronic kidney disease(CKD), and metabolic disorders [4, 5]. These diseases share pathological pathways, serving as comorbidities for each other and significantly impacting the risk of CVD occurrence and clinical outcomes. With the increasing prevalence of obesity and metabolic disorders, the incidence of cardiovascular-kidney-metabolic(CKM) syndrome continues to rise, along with the progression from preclinical CKM to clinical CVD stages [6]. Therefore, to curb the progression of CVDs and alleviate their significant clinical burden, the American Heart Association emphasizes the importance of conducting specialized research on the earliest preclinical stages (Stages 0–3) of CKM syndrome [7].

Current research indicates that the synergistic interaction between inflammation and insulin resistance (IR) is a fundamental mechanism driving residual CVD risk [8]. The C-reactive protein-triglyceride glucose index(CTI), first proposed by Ruan et al., is defined as a novel biomarker that can simultaneously reflect both IR and inflammation levels [9, 10]. Recent studies have revealed a complex threshold effect between cumulative CTI exposure and dynamic trajectories with CVD risk, particularly in populations with stage 0–3 CKM syndrome [11]. Subclinical inflammation and IR may exert synergistic effects, jointly activating pro-inflammatory pathways such as NF-κB in vascular endothelium, thereby promoting atherosclerotic plaque instability, endothelial injury, and thrombus formation [11, 12].

Obesity, as a chronic relapsing disease affecting multiple systems worldwide, is characterized primarily by the abnormal accumulation of adipose tissue [13–16]. Adipose tissue in different body regions exhibits varying cardiovascular risks due to functional differences, with visceral fat being recognized as closely associated with the development of metabolic syndrome and CVD through multiple pathways [17]. Therefore, the commonly used clinical obesity indicators have gradually shifted from Body Mass Index(BMI), which emphasizes body weight, to including waist circumference(WC) that can reflect visceral fat to some extent [18, 19]. Building upon this foundation and incorporating lipid levels that are crucial for the development of atherosclerosis and other CVDs, newly developed multiple obesity indices have demonstrated outstanding efficacy in predicting cardiometabolic risks [14]. However, these findings primarily focus on the combination of metabolic disorders and obesity states, failing to account for the critical role of inflammation in the pathogenesis of CVD. Furthermore, the existing evidence is primarily based on single-time-point measurements, overlooking the dynamic evolution characteristics of metabolic-inflammation-obesity burden and lacking longitudinal assessment of cumulative effects. Third, although it has been demonstrated that CTI-WHtR outperforms individual indicators in predicting stroke risk [20], it remains unclear which trajectory patterns of CTI obesity index combinations are most suitable for CVD risk assessment in the specific population of middle-aged and elderly individuals with CKM syndrome stages 0–3.

To address this critical research gap, this study systematically elucidates the association between CTI and its modified index with the risk of CVD incidence in patients at CKM stages 0–3, based on the China Health and Retirement Longitudinal Study(CHARLS) cohort data. Meanwhile, we quantified and evaluated the predictive value of cumulative exposure and dynamic trajectory changes of these composite indices on CVD risk in middle-aged and elderly populations. This study aims to establish a dynamic, multi-parameter risk assessment system to facilitate early intervention in the preclinical stage of CKM syndrome.

## Methods

### Study population

The data for this study were obtained from the CHARLS, which is a nationally representative prospective cohort study [21]. The study was initiated in 2011–2012 (Wave 1), initially recruiting approximately 17,000 participants. Follow-up surveys were conducted in 2013 (Wave 2), 2015 (Wave 3), 2018 (Wave 4), and 2020 (Wave 5). Notably, blood samples and anthropometric measurements were collected during Wave 1 and Wave 3, providing critical data for analyzing dynamic trajectory patterns of and its modified indices. The research complied with the Declaration of Helsinki and obtained approval from the Biomedical Ethics Review Board of Peking University (IRB 00001052–11015). All subjects signed informed consent forms before being enrolled in the study.

**Fig. 1:**
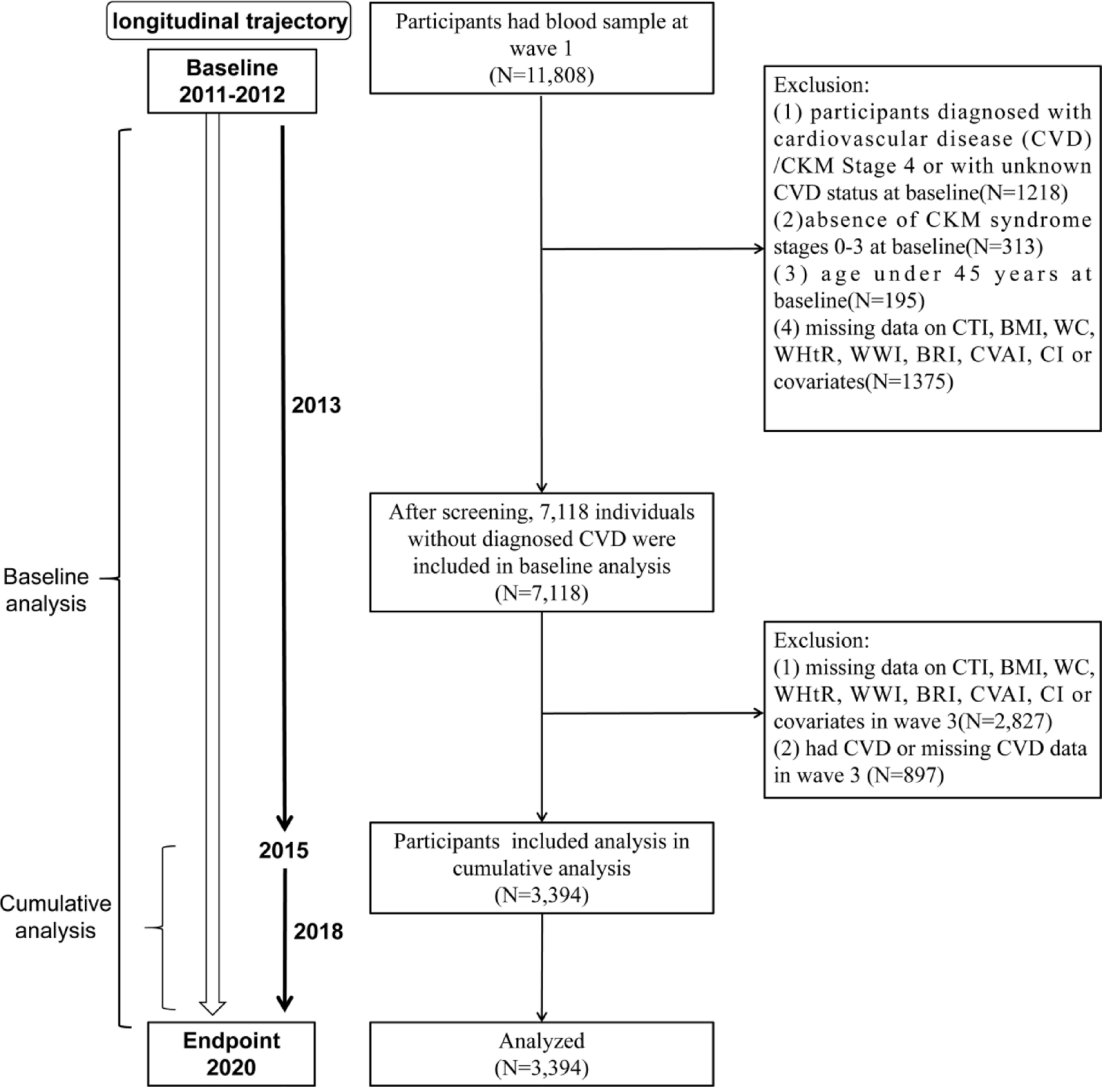
Selection process of the study population

In this study, participants with complete data on CTI and its modified indices between 2011 and 2012 were defined as the starting point of the cohort, and follow-up data were obtained in 2013, 2015, 2018, and 2020. The exclusion criteria are: (1) Age below 45 years at baseline; (2) absence of CKM stages 0–3 at baseline; (3) presence of CVD, heart disease, or stroke at baseline; (4) incomplete data on anthropometric, health-related, sociodemographic, or other biomarkers at baseline (Table S1). In the final analysis, we enrolled 7118 participants in the baseline analysis and 3,394 in the cumulative analysis. The detailed inclusion and exclusion criteria are shown in Fig. 1.

### Definition of CTI and its modified indices

The CTI index, a composite measure of C-reactive protein(CRP), triglycerides (TG), and fasting blood glucose (FBG), is a novel and effective indicator for assessing inflammation and IR. To better evaluate the synergistic effects of obesity and inflammatory glucose-lipid metabolic disorders, this study referred to the “multiplicative model” from previous methods [22, 23] and constructed a series of composite indices, with the formula as follows: CTI = 0.412 × Ln (CRP [mg/L]) + Ln (Tg [mg/dl] × FBG [mg/dl])/2; CTI-BMI = CTI × weight(kg)/height^2^ (m^2^); CTI-WC = CTI × WC(cm); CTI-WHtR = CTI × WC(cm) / height(cm); CTI-WWI = CTI × WC(cm) / sqrt(weight); CTI-BRI = CTI × 364.2–365.5 × sqrt(1 - (WC(cm)² / (2π)²) / (0.5 × height(cm))²); CTI-CVAI(Male) = CTI × (-267.93 + 0.68 × age + 0.03×BMI + 4.00 × WC(cm) + 22.00 × log10(Tg(mmol/L)) − 16.32 × HDL-C(mmol/L)); CTI-CVAI(Female) = CTI ×(− 187.32 + 1.71 × age + 4.23 × BMI + 1.12 × WC(cm) + 39.76 × log10(Tg(mmol/L) − 11.66 × HDL-C(mmol/L); CTI-CI = CTI × WC(cm)/ (10.9 * sqrt(weight(kg)/height(m)). Additionally, we calculated the cumulative values of these indices. The average exposure level was calculated by taking the mean of these indices at the two time points in 2012 and 2015, then multiplying it by the duration to determine the long-term cumulative exposure. This approach adheres to the methodology for cumulative metabolic indices in the CHARLS cohorts, which is consistent with the approach described by Ma [24] and Yang [25].

### Ascertainment of CKM syndrome 0–4 stages

According to the AHA Presidential Advisory Statement [4], the CKM syndrome stages are defined as follows: Stage 0: The absence of CKM syndrome risk factors. Stage 1: Excess adipose tissue(identified by either weight or abdominal obesity), and/or dysfunctional adipose tissue(manifested as impaired glucose tolerance and hyperglycemia). Stage 2: Presence of metabolic disorders, such as elevated TG, hypertension, diabetes, or metabolic syndrome, or moderate to high-risk CKD. Stage 3: Presence of subclinical CVD. In this study, CKM stage 3 subclinical CVD was defined as having a high predicted 10-year cardiovascular risk calculated based on the Framingham Risk Score, or the presence of CKD G4/G5. Stage 4: Clinical CVD, identified based on self-reported CVD history. The specific staging criteria for CKM stages 0 to 4 are provided in Table S2, and the diagnostic procedures for dyslipidemia, hypertension, diabetes, and metabolic syndrome are detailed in Table S3.

### Definition of clinical outcomes

Newly emerged CVD events were selected as the outcome measure. Outcome event occurrence was assessed by two standardized questions: “Have you been told by a doctor that you have been diagnosed with heart disease (including angina, heart attack, heart failure, coronary heart disease, or other heart problems)?” and “Have you been told by a doctor that you have been diagnosed with a stroke?”. Participants who had answered “yes” to one of these two questions were considered to have CVD.

### Data collection

Demographic data, lifestyle habits, comorbidities, physical and laboratory tests of participants were gathered. Demographic data: age, gender, residence place, marital status, and education levels. Lifestyle habits: smoking status and drinking status. Comorbidities: hypertension, dyslipidemia and diabetes. Physical examinations: BMI, WC, height, weight, systolic blood pressure (SBP), and diastolic blood pressure (DBP). Laboratory tests: CRP, FBG, glycated hemoglobin A1c (HbA1c), TG, total cholesterol (TC), high-density lipoprotein cholesterol (HDL-C), low-density lipoprotein cholesterol (LDL-C), serum creatinine(Scr), blood urea nitrogen(BUN), uric acid(UA), and estimated glomerular filtration rate (eGFR).

### Statistical analysis

Baseline characteristics were presented as mean ± standard deviation (SD) for continuous variables with normal distribution, or medians (interquartile ranges [IQRs]) for skewed data. Categorical variables were expressed as frequencies and percentages. Group differences were evaluated using one-way ANOVA, the Kruskal-Wallis test, or the chi-square test as appropriate. The longitudinal association between CTI-related indices and incident CVD was evaluated using Cox proportional hazards regression models. Multivariate models were constructed with incremental adjustment for confounders. Notably, to avoid over-adjustment bias, components inherently used to define the CKM syndrome framework (i.e., baseline diabetes and dyslipidemia) were excluded from the adjusted covariates to ensure the independent prognostic value of the indices was accurately captured. The Crude Model was unadjusted; Model 1 was adjusted for age, sex, marital status, education, eGFR, smoke status, drink status, and residence place; and Model 2 was further adjusted for BUN, UA, LDL-C, HDL-C, and HbA1c. Multicollinearity diagnostics confirmed all variance inflation factors (VIF) remained below 5, indicating that the interdependencies among covariates were negligible (Tables S4, and S5). The proportional hazards assumption was tested using Schoenfeld residuals, and no potential violation was observed (global *P* > 0.05) (Tables S6, and S7).

The trajectory of cumulative CTI and its derivative indices was determined through K-means cluster analysis. It is an unsupervised machine learning method for data clustering analysis [26]. The optimal number of clusters, determined using the elbow method, was found to be k = 3 after 100 iterations. Cluster 1 showed stable low levels, Class 2 maintained moderate levels, and Class 3 displayed high levels (Fig. S1). The dose-response relationships between CTI indices (both baseline and cumulative) and CVD risk were characterized using restricted cubic splines (RCS) with three knots at the 25th, 50th, and 75th percentiles.

The incremental predictive value of the modified indices compared with CTI was assessed through time-dependent Receiver Operating Characteristic (ROC) curve analysis. We employed the Nearest Neighbor Estimation (NNE) method to calculate time-dependent Areas Under the Curve (AUC) across various follow-up time points. To statistically compare the discriminative ability between the modified indices and the reference CTI, a Bootstrap resampling procedure (500 iterations) was implemented to derive the empirical distribution of AUC differences and calculate two-sided P-values. Furthermore, the continuous Net Reclassification Improvement (cNRI) and Integrated Discrimination Improvement (IDI) were calculated to quantify the improvement in risk stratification.

To distinguish the relative contributions of individual components within the composite indices, Weighted Quantile Sum (WQS) regression was utilized to estimate the weighted impact of glycemic, lipidemic, inflammatory, and adiposity parameters on CVD hazard. Frailty is a clinically complex condition closely associated with advancing age, characterized by a decline in physiological function across multiple organ systems. Previous studies have confirmed that frailty is associated with various adverse outcomes in older adults, including CVD, stroke, falls, and cognitive decline. Therefore, frailty (represented by the frailty index(FI)) was selected as a potential mediating factor for the associations of CTI and its modified indices and incident CVD. The quantitative method of the FI is presented in Table S8.

A sensitivity analysis was conducted on the data to verify the robustness of the results. Firstly, we compared baseline characteristics between the included and excluded participants to assess selection bias. Secondly, we reassessed the correlation between CTI and its modified indices with incident CVD using the IQR of the indicators. Furthermore, recognizing the high mortality rate in the aging cohort, we implemented the Fine-Gray proportional sub-distribution hazards model to account for deaths not caused by CVD as competing events. The cumulative incidence function (CIF) for CVD was estimated, and Gray’s test was used to compare the between-group difference in cumulative CVD incidence.

All analyses were performed using R software (version 4.3.1). Statistical significance was defined as a two-sided *P* < 0.05.

## Results

### Baseline characteristics

#### Baseline characteristics of study participants stratified by the occurrence of CVD

Table 1 presents the baseline demographic and clinical characteristics of the 7,118 participants (mean age 58.52 ± 9.48 years; 54.09% female) included in the final analysis, stratified by the occurrence of CVD. Over the follow-up period, 1,617 individuals (22.71%) developed incident CVD. Compared to those who remained CVD-free, participants with incident CVD were significantly older (59.66 ± 8.89 vs. 58.18 ± 9.62 years, *P* < 0.0001) and exhibited a heavier burden of metabolic comorbidities, including higher prevalence of hypertension (64.01% vs. 50.83%), diabetes (17.32% vs. 13.60%), and dyslipidemia (44.59% vs. 35.98%) (all *P* < 0.0001). Anthropometric assessments revealed significantly higher BMI and WC in the CVD group. Biochemically, individuals with incident CVD were characterized by a more pro-inflammatory and dysmetabolic state, evidenced by elevated levels of CRP, FBG, HbA1c, and TG, alongside significantly lower HDL-C and eGFR levels. Notably, the CTI index and all its obesity-modified derivatives were significantly higher in the CVD group compared to the non-CVD group.

**Table 1:** Baseline characteristics of the study population based on new-onset CVD

Variable	Total (n = 7118)	No (n = 5501)	Yes (n = 1617)	*p* value
Sex, n (%)				< 0.001
female	3850(54.09)	2926(53.19)	924(57.14)	
male	3268(45.91)	2575(46.81)	693(42.86)	
Age, years	58.52 ± 9.48	58.18 ± 9.62	59.66 ± 8.89	< 0.001
Educational level, n (%)				0.550
above junior high school	707( 9.93)	535( 9.73)	172(10.64)	
illiterate	3383(47.53)	2618(47.59)	765(47.31)	
junior high school and below	3028(42.54)	2348(42.68)	680(42.05)	

Marital status, n (%)				0.500
married	6321(88.80)	4877(88.66)	1444(89.30)	
other	797(11.20)	624(11.34)	173(10.70)	
Residence place, n (%)				0.550
rural	4672(65.64)	3600(65.44)	1072(66.30)	
urban	2446(34.36)	1901(34.56)	545(33.70)	
Smoke, n (%)				< 0.001
current	2140(30.06)	1698(30.87)	442(27.33)	
ever	578( 8.12)	413( 7.51)	165(10.20)	
never	4400(61.82)	3390(61.63)	1010(62.46)	

Drink, n (%)				0.010
no	4701(66.04)	3590(65.26)	1111(68.71)	
yes	2417(33.96)	1911(34.74)	506(31.29)	
Hypertension, n (%)				< 0.001
no	3287(46.18)	2705(49.17)	582(35.99)	
yes	3831(53.82)	2796(50.83)	1035(64.01)	
Diabetes, n (%)				< 0.001
no	6090(85.56)	4753(86.40)	1337(82.68)	
yes	1028(14.44)	748(13.60)	280(17.32)	

Dyslipidemian, n (%)				< 0.001
no	4418(62.07)	3522(64.02)	896(55.41)	
yes	2700(37.93)	1979(35.98)	721(44.59)	
BMI(kg/m2)	23.48 ± 3.81	23.27 ± 3.71	24.19 ± 4.02	< 0.001
WC(cm)	84.09 ± 12.15	83.50 ± 11.87	86.09 ± 12.87	< 0.001
SBP(mmHg)	129.63 ± 20.99	128.59 ± 20.66	133.15 ± 21.70	< 0.001
DBP(mmHg)	75.53 ± 12.00	75.04 ± 11.88	77.21 ± 12.29	< 0.001
HbA1c(%)	5.27 ± 0.79	5.24 ± 0.74	5.36 ± 0.93	< 0.001
HDL-C(mg/dL)	51.90 ± 15.18	52.26 ± 15.17	50.68 ± 15.14	< 0.001
LDL-C(mg/dL)	3.04 ± 0.90	3.01 ± 0.90	3.11 ± 0.92	< 0.001
Scr(mg/dL)	0.78 ± 0.24	0.78 ± 0.25	0.77 ± 0.18	0.320
BUN(mg/dL)	15.66 ± 4.52	15.72 ± 4.55	15.48 ± 4.42	0.060
UA(mg/dL)	4.41 ± 1.23	4.42 ± 1.23	4.38 ± 1.23	0.350
FBG(mg/dL)	108.89 ± 33.32	108.02 ± 32.07	111.84 ± 37.12	< 0.001
TG(mg/dL)	127.07 ± 91.37	125.10 ± 92.08	133.76 ± 88.64	< 0.001
CRP(mg/dL)	2.63 ± 7.42	2.64 ± 7.75	2.59 ± 6.15	0.780
eGFR(ml/min·1.73m^2^)	96.49 ± 14.01	96.78 ± 14.06	95.50 ± 13.77	< 0.010

CKM, n (%)				< 0.001
0	560( 7.87)	488( 8.87)	72( 4.45)	
1	1307(18.36)	1070(19.45)	237(14.66)	
2	2736(38.44)	2111(38.37)	625(38.65)	
3	2515(35.33)	1832(33.30)	683(42.24)	
CTI	4.73 ± 0.57	4.71 ± 0.57	4.81 ± 0.57	< 0.001
CTI-BMI	111.63 ± 25.55	110.04 ± 24.83	117.01 ± 27.17	< 0.001
CTI-WC	399.44 ± 84.77	394.58 ± 82.85	415.96 ± 89.07	< 0.001
CTI-WHtR	2.53 ± 0.54	2.50 ± 0.53	2.64 ± 0.57	< 0.001
CTI-WWI	52.31 ± 9.42	51.92 ± 9.33	53.64 ± 9.60	< 0.001
CTI-BRI	19.77 ± 8.12	19.29 ± 7.89	21.41 ± 8.64	< 0.001
CTI-CVAI	444.97 ± 241.23	429.26 ± 236.52	498.40 ± 249.39	< 0.001
CTI-CI	6.02 ± 1.06	5.98 ± 1.05	6.18 ± 1.09	< 0.001

Continuous variables are expressed as Mean ± SD or Median (IQR), categorical variables are expressed as number (percent). BMI: body mass index; SBP: Systolic blood pressure; DBP: Diastolic blood pressure; HbA1c: Glycosylated Hemoglobin, Type A1C; FBG: fast blood glucose; HDL-C: high density lipoprotein cholesterol; TG: total cholesterol; LDL-C: low density lipoprotein cholesterol; Scr: serum creatinine; BUN: blood urea nitrogen; UA: uric acid; eGFR: estimated glomerular filtration rate; CKM: cardiovascular-kidney-metabolic; CTI: C-reactive protein-triglyceride-glucose index; CVD: cardiovascular disease; WC: waist circumference; WHtR: Waist-to-Height Ratio; WWI: Weight-adjusted waist index; BRI: Body Roundness Index; CVAI: Chinese Visceral Adiposity Index; CI: C-index

#### Baseline characteristics of study participants stratified by CKM syndrome stages

Stratification by CKM syndrome stages (Stages 0–3) revealed a striking and progressive escalation in both cardiovascular risk factors and clinical outcomes (Table S9). Most notably, the prevalence of incident CVD increased more than twofold from Stage 0 (12.86%) to Stage 3 (27.16%) (*P* < 0.0001). As participants progressed from preclinical CKM (Stage 0) to advanced subclinical stages (Stage 3), the physiological parameters exhibited an overall deteriorating trend, characterized by advancing age, higher SBP and DBP, and worsening renal function (evidenced by increasing Scr, BUN, and UA levels alongside declining eGFR). Metabolic markers, specifically FBG, TG, and HbA1c, showed step-wise increments across the stages. Furthermore, systemic inflammation, represented by CRP, remained relatively stable in early CKM stages but increased markedly in advanced stages. Correspondingly, the CTI index and its modified indicators exhibited a significant upward gradient across stages.

### Association of CTI and its modified indices with CVDs

#### Association of baseline CTI and its modified indices with incident CVD

In the multivariate Cox regression analysis, when CTI and its modified indices were analyzed as continuous variables (per SD), the results showed significant positive associations between all indices and CVD. After adjusting for demographic and clinical confounders, compared to CTI (HR 1.13, 95%CI 1.07–1.20, *P* < 0.001), CTI-WC (HR 1.23, 95%CI 1.16–1.30, *P* < 0.001), CTI-WHtR (HR 1.00, 95%CI 1.00–1.00, *P* < 0.001), CTI-WWI (HR 1.11, 95%CI 1.05–1.18, *P* < 0.001), CTI-BRI (HR 1.19, 95%CI 1.13–1.26, *P* < 0.001), CTI-BMI (HR 1.24, 95%CI 1.18–1.31, *P* < 0.001) and CTI-CI (HR 1.13, 95%CI 1.06–1.19, *P* < 0.001), the CTI-CVAI demonstrated the strongest correlation with CVD, with each 1-SD increase associated with a 27% higher incidence of CVD (HR 1.27, 95%CI 1.19–1.35, *P* < 0.001). We subsequently categorized the CTI and modified CTI indices into quartiles, revealing a distinct dose-response relationship, with CVD risk rising progressively across successive quartiles (P for trend < 0.001) (Table 2) (Tables S10–11). Subgroup analysis showed that in the early to mid-stage CKM (stage 0–2) populations, the associations between CTI and its modified indices and incident CVD were amplified (*P* < 0.001). In contrast, CTI-CI and CTI-WWI lost statistical correlation with the incidence of CVD in advanced CKM (stage 3) populations (*P* > 0.05).

**Table 2:** Associations between CTI and its related indices and CVD risk in CKM syndrome stage 0–3

	Crude model		Model 1		Model 2	
	95%CI	P	95%CI	P	95%CI	P
Continues CTI(per SD)	1.2(1.15,1.26)	< 0.0001	1.17(1.12,1.23)	< 0.0001	1.13(1.07,1.19)	< 0.0001
*CTI*						
Q1	ref		ref		ref	
Q2	1.3(1.12,1.51)	< 0.001	1.24(1.07,1.44)	0.005	1.21(1.04,1.40)	0.02
Q3	1.56(1.35,1.80)	< 0.0001	1.46(1.26,1.69)	< 0.0001	1.4(1.20,1.63)	< 0.0001
Q4	1.79(1.55,2.06)	< 0.0001	1.66(1.44,1.92)	< 0.0001	1.52(1.29,1.79)	< 0.0001
P for trend		< 0.0001		< 0.0001		< 0.0001
Continues CTI- BMI (per SD)	1.25(1.19,1.30)	< 0.0001	1.25(1.19,1.30)	< 0.0001	1.24(1.18,1.30)	< 0.0001
*CTI-BMI*						
Q1	ref		ref		ref	
Q2	1.23(1.06,1.43)	0.01	1.26(1.08,1.47)	0.003	1.25(1.07,1.46)	0.005
Q3	1.41(1.22,1.64)	< 0.0001	1.44(1.24,1.67)	< 0.0001	1.42(1.21,1.66)	< 0.0001
Q4	1.9(1.65,2.19)	< 0.0001	1.94(1.68,2.25)	< 0.0001	1.89(1.61,2.23)	< 0.0001
P for trend		< 0.0001		< 0.0001		< 0.0001
Continues CTI-WC (per SD)	1.27(1.21,1.34)	< 0.0001	1.25(1.19,1.31)	< 0.0001	1.23(1.16,1.30)	< 0.0001

CTI-WC						
Q1	ref		ref		ref	
Q2	1.27(1.09,1.48)	0.002	1.24(1.06,1.44)	0.01	1.23(1.05,1.43)	0.01
Q3	1.59(1.37,1.84)	< 0.0001	1.52(1.31,1.76)	< 0.0001	1.5(1.28,1.75)	< 0.0001
Q4	1.93(1.68,2.23)	< 0.0001	1.84(1.59,2.12)	< 0.0001	1.77(1.50,2.08)	< 0.0001
P for trend		< 0.0001		< 0.0001		< 0.0001
Continues CTI-WHtR (per SD)	1(1.00,1.00)	< 0.0001	1(1.00,1.00)	< 0.0001	1(1.00,1.00)	< 0.0001
CTI-WHtR						
Q1	ref		ref		ref	
Q2	1.25(1.07,1.46)	0.004	1.24(1.06,1.44)	0.01	1.23(1.05,1.43)	0.01
Q3	1.61(1.39,1.87)	< 0.0001	1.54(1.33,1.78)	< 0.0001	1.5(1.29,1.75)	< 0.0001
Q4	1.96(1.70,2.26)	< 0.0001	1.83(1.58,2.12)	< 0.0001	1.74(1.48,2.06)	< 0.0001
P for trend		< 0.0001		< 0.0001		< 0.0001
Continues CTI-WWI (per SD)	1.23(1.17,1.29)	< 0.0001	1.16(1.10,1.22)	< 0.0001	1.11(1.05,1.18)	< 0.001
CTI-WWI						
Q1	ref		ref		ref	
Q2	1.31(1.12,1.52)	< 0.001	1.24(1.07,1.44)	0.01	1.22(1.04,1.42)	0.01
Q3	1.57(1.36,1.82)	< 0.0001	1.45(1.25,1.68)	< 0.0001	1.37(1.18,1.60)	< 0.0001
Q4	1.85(1.61,2.14)	< 0.0001	1.63(1.41,1.89)	< 0.0001	1.48(1.26,1.75)	< 0.0001
P for trend		< 0.0001		< 0.0001		< 0.0001
Continues CTI-BRI (per SD)	1.26(1.20,1.32)	< 0.0001	1.22(1.17,1.29)	< 0.0001	1.19(1.13,1.26)	< 0.0001

CTI-BRI						
Q1	ref		ref		ref	
Q2	1.19(1.02,1.39)	0.02	1.2(1.03,1.40)	0.02	1.18(1.01,1.38)	0.04
Q3	1.5(1.29,1.73)	< 0.0001	1.46(1.26,1.69)	< 0.0001	1.41(1.21,1.65)	< 0.0001
Q4	1.92(1.67,2.21)	< 0.0001	1.82(1.57,2.11)	< 0.0001	1.72(1.46,2.02)	< 0.0001
P for trend		< 0.0001		< 0.0001		< 0.0001
Continues CTI-CVAI (per SD)	1.32(1.26,1.38)	< 0.0001	1.26(1.20,1.33)	< 0.0001	1.27(1.19,1.35)	< 0.0001
CTI-CVAI						
Q1	ref		ref		ref	
Q2	1.33(1.14,1.56)	< 0.001	1.26(1.08,1.47)	0.004	1.26(1.07,1.48)	0.005
Q3	1.68(1.45,1.95)	< 0.0001	1.54(1.32,1.79)	< 0.0001	1.55(1.31,1.83)	< 0.0001
Q4	2.14(1.85,2.47)	< 0.0001	1.89(1.63,2.19)	< 0.0001	1.9(1.59,2.27)	< 0.0001
P for trend		< 0.0001		< 0.0001		< 0.0001
Continues CTI-CI (per SD)	1.23(1.17,1.30)	< 0.0001	1.18(1.12,1.24)	< 0.0001	1.13(1.06,1.19)	< 0.0001

CTI-CI						
Q1	ref		ref		ref	
Q2	1.24(1.07,1.45)	0.005	1.18(1.01,1.37)	0.03	1.16(1.00,1.35)	0.06
Q3	1.58(1.37,1.83)	< 0.0001	1.46(1.26,1.69)	< 0.0001	1.4(1.20,1.63)	< 0.0001
Q4	1.84(1.60,2.13)	< 0.0001	1.64(1.41,1.89)	< 0.0001	1.5(1.28,1.77)	< 0.0001
P for trend		< 0.0001		< 0.0001		< 0.0001
Columative CTI (per SD)	1.26(1.17,1.35)	< 0.0001	1.25(1.16,1.35)	< 0.0001	1.18(1.07,1.29)	< 0.001
Cluster CTI						
low	ref		ref		ref	
medium	1.48(1.22,1.79)	< 0.0001	1.45(1.20,1.75)	< 0.001	1.33(1.09,1.62)	0.004
high	1.82(1.47,2.25)	< 0.0001	1.79(1.44,2.22)	< 0.0001	1.49(1.16,1.91)	0.002
P for trend		< 0.0001		< 0.0001		0.001
Columative CTI-BMI (per SD)	1.3(1.21,1.40)	< 0.0001	1.34(1.24,1.44)	< 0.0001	1.29(1.18,1.41)	< 0.0001
Cluster BMI						
low	ref		ref		ref	
medium	1.44(1.20,1.74)	< 0.001	1.5(1.24,1.81)	< 0.0001	1.39(1.14,1.70)	0.001
high	1.96(1.59,2.42)	< 0.0001	2.09(1.68,2.59)	< 0.0001	1.82(1.42,2.32)	< 0.0001
P for trend		< 0.0001		< 0.0001		< 0.0001
Columative CTI-WC (per SD)	1.31(1.22,1.42)	< 0.0001	1.32(1.22,1.43)	< 0.0001	1.26(1.15,1.38)	< 0.0001

Cluster CTI-WC						
low	ref		ref		ref	
medium	1.35(1.11,1.64)	0.002	1.35(1.11,1.65)	0.002	1.28(1.04,1.57)	0.02
high	2.18(1.77,2.68)	< 0.0001	2.19(1.77,2.70)	< 0.0001	1.94(1.52,2.48)	< 0.0001
P for trend		< 0.0001		< 0.0001		< 0.0001
Columative CTI-WHtR (per SD)	1.33(1.23,1.43)	< 0.0001	1.31(1.21,1.42)	< 0.0001	1.25(1.14,1.37)	< 0.0001
Cluster CTI-WHtR						
low	ref		ref		ref	
medium	1.5(1.24,1.83)	< 0.0001	1.49(1.22,1.82)	< 0.0001	1.39(1.13,1.71)	0.002
high	2.08(1.68,2.57)	< 0.0001	2.04(1.63,2.54)	< 0.0001	1.77(1.38,2.27)	< 0.0001
P for trend		< 0.0001		< 0.0001		< 0.0001
Columative CTI-WWI (per SD)	1.29(1.19,1.39)	< 0.0001	1.25(1.15,1.35)	< 0.0001	1.16(1.06,1.28)	0.002

Cluster CTI-WWI						
low	ref		ref		ref	
medium	1.38(1.11,1.72)	0.004	1.34(1.08,1.68)	0.01	1.24(0.99,1.56)	0.06
high	2.02(1.60,2.53)	< 0.0001	1.88(1.48,2.38)	< 0.0001	1.59(1.22,2.06)	< 0.001
P for trend		< 0.0001		< 0.0001		< 0.001
Columative CTI-BRI (per SD)	1.32(1.23,1.42)	< 0.0001	1.31(1.21,1.41)	< 0.0001	1.24(1.14,1.35)	< 0.0001
Cluster CTI-BRI						
low	ref		ref		ref	
medium	1.4(1.16,1.69)	< 0.001	1.4(1.16,1.70)	< 0.001	1.3(1.06,1.59)	0.01
high	2.19(1.78,2.70)	< 0.0001	2.16(1.73,2.69)	< 0.0001	1.9(1.49,2.43)	< 0.0001
P for trend		< 0.0001		< 0.0001		< 0.0001
Columative CTI-CVAI (per SD)	1.35(1.25,1.45)	< 0.0001	1.32(1.22,1.43)	< 0.0001	1.28(1.16,1.41)	< 0.0001

Cluster CTI-CVAI						
low	ref		ref		ref	
medium	1.73(1.41,2.12)	< 0.0001	1.67(1.36,2.06)	< 0.0001	1.61(1.29,2.00)	< 0.0001
high	2.39(1.92,2.98)	< 0.0001	2.29(1.83,2.87)	< 0.0001	2.1(1.62,2.73)	< 0.0001
P for trend		< 0.0001		< 0.0001		< 0.0001
Columative CTI-CI (per SD)	1.29(1.19,1.39)	< 0.0001	1.26(1.16,1.36)	< 0.0001	1.18(1.07,1.29)	< 0.001

Cluster CTI-CI						
low	ref		ref		ref	
medium	1.39(1.12,1.74)	0.003	1.36(1.09,1.70)	0.01	1.26(1.01,1.59)	0.04
high	1.97(1.56,2.48)	< 0.0001	1.84(1.46,2.33)	< 0.0001	1.55(1.20,2.01)	< 0.001
P for trend		< 0.0001		< 0.0001		< 0.001

HR Hazard Ratio, CI Confidence Interval

Crude model: unadjusted for covariates;

Model 1: age, sex, marital status, education, eGFR, smoke status, drink status, residence place;

Model 2: age, sex, marital status, education, eGFR, smoke status, drink status, residence place, BUN, UA, LDL-C, HDL-C, HbA1c

CTI: C-reactive protein-triglyceride-glucose index; CKM: cardiovascular–kidney–metabolic; CVD: cardiovascular disease; WC: waist circumference; WHtR: Waist-to-Height Ratio; WWI: Weight-adjusted waist index; BRI: Body Roundness Index; CVAI: Chinese Visceral Adiposity Index; CI: C-index

#### Association of cumulative CTI and its modified indices with incident CVD

To capture the long-term impact of metabolic and inflammatory burden, we further examined the associations of cumulative exposure and dynamic trajectory clusters of CTI and its modified indices with CVD risk. Cumulative exposure to modified CTI indices significantly elevated the risk of CVD in the total population. In the fully adjusted Model 2, continuous variable analysis demonstrated that cumulative CTI-BMI (HR per SD: 1.29, 95% CI 1.18–1.41, *P* < 0.001), cumulative CTI-WC (HR per SD: 1.26, 95% CI 1.15–1.38, *P* < 0.001), cumulative CTI-WHtR (HR per SD: 1.25, 95% CI 1.14–1.37, *P* < 0.001), cumulative CTI-WWI (HR per SD: 1.16, 95% CI 1.06–1.28, *P* < 0.001), cumulative CTI-BRI (HR per SD: 1.24, 95% CI 1.14–1.35, *P* < 0.001), cumulative CTI-CVAI (HR per SD: 1.28, 95% CI 1.16–1.41, *P* < 0.001), and cumulative CTI-CI (HR per SD: 1.18, 95% CI 1.07–1.29, *P* < 0.001)were significantly associated with an increased hazard of incident CVD (Table 2) (Tables S10–11). Dynamic trajectory patterns further illustrated the longitudinal risk of sustained high exposure. Compared to the low trajectory cluster, participants in the high trajectory group of cumulative CTI-BMI exhibited a 82% increased risk of CVD (HR 1.82, 95% CI 1.42–2.32, *P* < 0.001; P for trend < 0.001). Notably, the high trajectory cluster of cumulative CTI-CVAI was associated with a nearly two-fold increase in CVD risk (HR 2.10, 95% CI 1.62–2.73, *P* < 0.001). Subgroup analyses confirmed the persistence of these longitudinal associations across CKM stages. In the CKM stage 0–2 subgroup, sustained high exposure to CTI and its modified indices were all significantly associated with an increased risk of CVD. Among them, CTI-CVAI (high trajectory) showed the strongest correlation with increased CVD risk (HR: 2.19, 95% CI 1.54–3.11, *P* < 0.001). In the CKM stage 3 subgroup, although continuous cumulative exposure for some indices showed attenuated significance, the high trajectory clusters for CTI-WC (HR 1.70, 95% CI 1.15–2.51, *P* = 0.010), CTI-BRI (HR 1.68, 95% CI 1.15–2.47, *P* = 0.010) and CTI-CVAI (HR 1.73, 95% CI 1.14–2.63, *P* = 0.010) remained strongly associated with higher CVD risk (Table 2) (Tables S10–11).

### Dose-response relationships between CTI-related indices and incident CVD

**Fig. 2:**
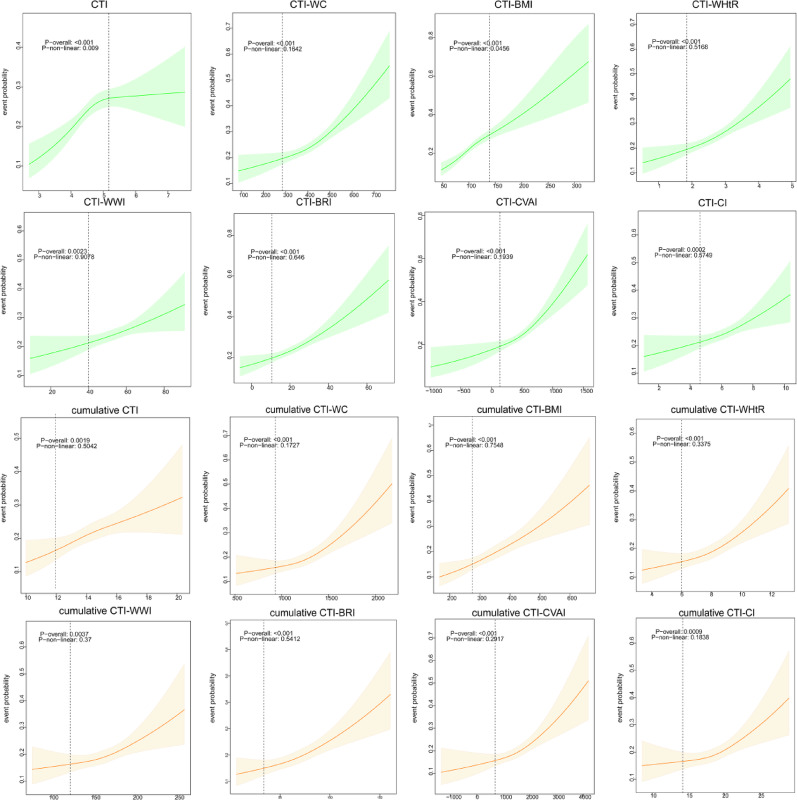
Analysis of restricted cubic spline regression

To further elucidate the nature of the associations, restricted cubic spline (RCS) regression was employed to evaluate the dose-response relationships between CTI-related indices and incident CVD (Fig. 2). Consistent with the Cox proportional hazards models, all analyses were adjusted for the full set of covariates in Model 2. Baseline CTI-related indices exhibited distinct dose-response patterns. Notably, both the CTI index(P _overall_ < 0.001, P _non−linear_ = 0.009) and CTI-BMI(P _overall_ < 0.001, P _non−linear_ = 0.046) showed significant non-linear associations with CVD risk, characterized by a rapid escalation in hazard at lower index values that tended to plateau at higher levels(Table S12–13). In contrast, other modified indices that reached statistical significance in the Cox models—including CTI-WC, CTI-WHtR, CTI-WWI, CTI-BRI, CTI-CVAI, and CTI-CI—demonstrated robust and monotonic linear associations with the hazard of CVD (all P _overall_ < 0.0001, P _non−linear_ > 0.05). For these modified metrics, the risk of incident CVD increased steadily and proportionally across the entire range of exposure values.

Unlike the baseline metrics, cumulative exposure to both the unmodified CTI and its obesity-modified derivatives showed purely linear relationships with CVD risk (all P _non−linear_ > 0.05). Specifically, cumulative CTI-BMI (P _overall_ < 0.001), cumulative CTI-WC (P _overall_ < 0.001), cumulative CTI-WHtR (P _overall_ < 0.001), cumulative CTI-WWI (P _overall_ = 0.004), cumulative CTI-BRI (P _overall_ < 0.001), cumulative CTI-CVAI (P _overall_ < 0.001), and cumulative CTI-CI (P _overall_ = 0.0009) exhibited the significant linear trends (Fig. 2).

### Comparative predictive performance of CTI and its modified indices

**Fig. 3:**
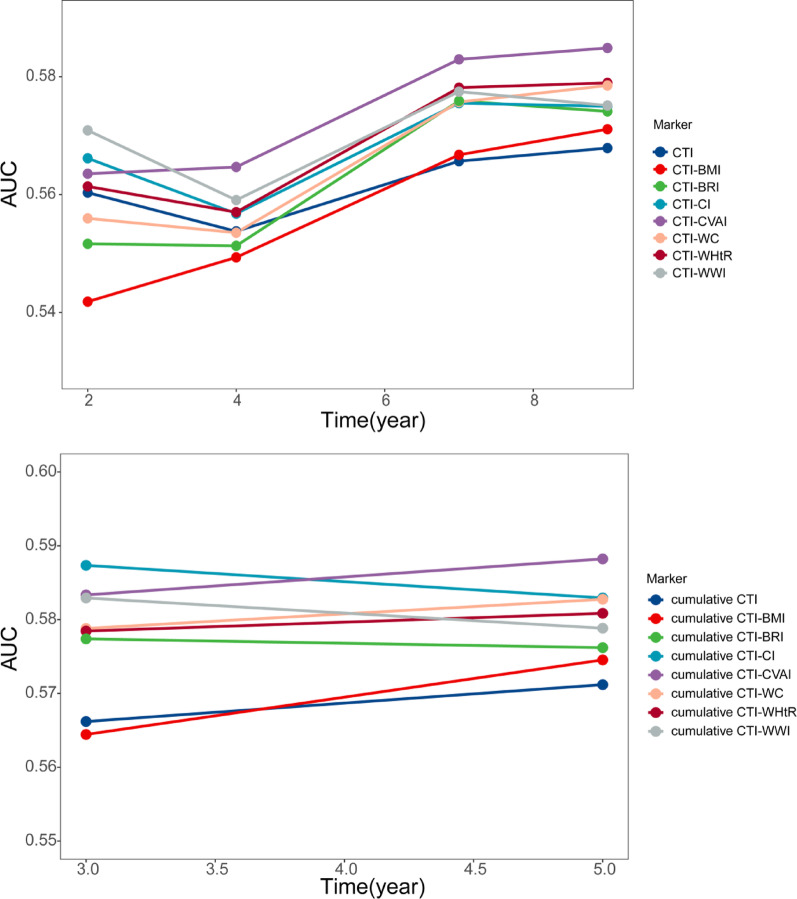
Time-dependent AUC changes of CTI and its combined obesity-related indices during follow-up. The upper panel shows the dynamic AUC changes of single baseline-measured CTI and its combined indices (CTI-BMI, CTI-WC, CTI-WHtR, CTI-BRI, CTI-CVAI, CTI-CI, CTI-WWI) at 2, 4, 7, and 9 years of follow-up. The lower panel presents the dynamic AUC changes of cumulative-measured CTI and its combined indices at 3.0 and 5.0 years of follow-up

To assess the incremental predictive value of obesity-modified CTI indices, we compared their discriminative ability and risk reclassification improvement against the original CTI (Fig. 3). For discrimination, the AUC increments for all modified indices, including CTI-CVAI (AUC difference: 0.017, *P* = 0.076), failed to reach statistical significance. However, CTI-CVAI significantly improved risk classification, providing a cNRI of 0.126 and an Integrated Discrimination Improvement (IDI) of 0.026 (both *P* < 0.001). Similarly, CTI-BMI, CTI-WC, and CTI-WHtR also demonstrated significant improvements in both cNRI and IDI (all *P* < 0.05), whereas CTI-CI and CTI-WWI showed no significant reclassification benefits (Tables S14–15).

Stratified analyses by CKM stage revealed statistically significant differences in predictive increments. In CKM Stage 0, compared with CTI, a significant improvement in overall discriminative ability was only observed for CTI-WWI at the 7-year follow-up (IDI = 0.044, *P* = 0.044). Conversely, in the high-risk CKM Stage 3 cohort, CTI-CVAI and CTI-WC significantly improved both discriminative ability and reclassification at the 9-year follow-up (*P* < 0.05).

Regarding cumulative exposure (5-year follow-up), predictive increments were only significant for cumulative CTI-CVAI in reclassification metrics. While its AUC change was not significant (difference: 0.017, *P* = 0.168), cumulative CTI-CVAI significantly improved reclassification over cumulative CTI (cNRI: 0.093, *P* = 0.040; IDI: 0.024, *P* = 0.016). Other cumulative indices showed no statistically significant improvements in AUC, cNRI, or IDI (Tables S16–17).

### WQS analysis

**Fig. 4:**
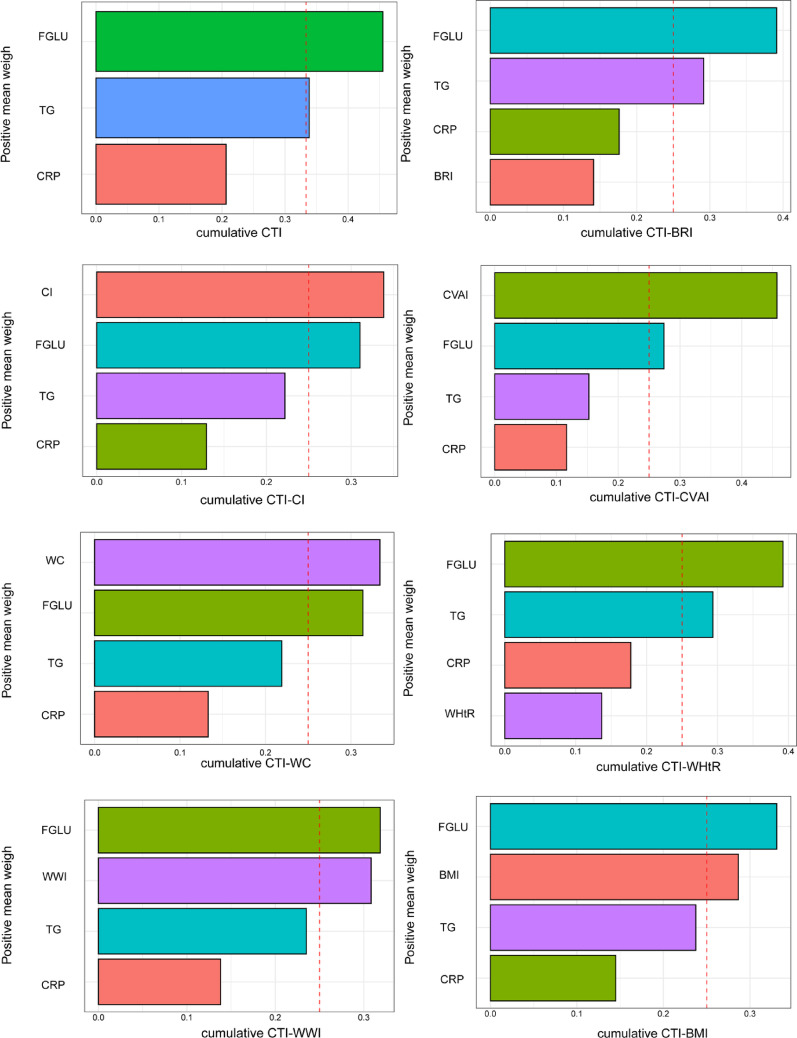
Relative weights of fasting blood glucose, C-reactive protein, triglycerides and obesity index contributing to CVD risk via WQS regression

To directly address the relative importance of inflammation versus metabolic dysfunction in driving cardiovascular risk, we applied WQS regression to estimate the specific weight of each component within the cumulative exposure models (Fig. 4). In the unmodified cumulative CTI model, FBG emerged as the most heavily weighted component, accounting for the largest proportion of the risk association, followed sequentially by TG and CRP. When the obesity-related parameters were integrated into the WQS models, they exhibited diverse weighting profiles. Most notably, in the cumulative CTI-CVAI model, the CVAI component overwhelmingly dominated the risk association, securing the highest positive mean weight and substantially attenuating the relative contributions of FBG, TG, and CRP. Similarly, WC and the CI assumed the top weighted positions in their respective models. In contrast, components like BMI and WHtR shared more balanced risk contributions alongside FBG.

### Mediation analysis of frailty in the association between CTI indices and CVD

To further elucidate the pathophysiological pathways, we performed causal mediation analysis to evaluate whether frailty serves as a link between metabolic-inflammatory burden and incident CVD. The results indicate that frailty emerged as a significant mediator for all CTI-related indices. In the model for the unmodified CTI index, the total effect was significant (estimate = 0.0189, *P* < 0.001), with an ADE of 0.0152 (*P* = 0.012) and an ACME of 0.0038 (*P* < 0.001). Among the obesity-modified indices, the proportion of the total effect mediated by frailty varied significantly. Overall, it explained 5.8 to 32.0% of the association between elevated CTI and its modification index with CVD events (Fig. S2).

### Sensitivity analyses

To ensure the robustness of our primary findings, we performed several comprehensive sensitivity analyses. Firstly, we compared the baseline characteristics between the excluded and included participants. Given the large sample size, minor variations in several variables achieved statistical significance; however, the effect sizes for the magnitude of these differences were negligible (Table S18). Secondly, we evaluated the impact of exposure increments. When analyzing baseline CTI and its modified indices as continuous variables per interquartile range (IQR) increment, the positive associations with incident CVD remained statistically significant across all models (all *P* < 0.05) (Tables S19–20). Finally, to strictly account for the potential bias of mortality in this aging cohort, we employed the Fine-Gray proportional sub-distribution hazards model. The competing risk analyses objectively confirmed that all indices maintained robust and highly significant associations with incident CVD, independent of the competing risk of non-CVD death. Specifically, the optimal obesity-modified indices exhibited superior predictive strength compared to the original CTI. For instance, the highest quartile of CTI-CVAI yielded a subHR of 1.85 (95% CI 1.56–2.19, *P* < 0.0001), and CTI-BMI yielded a subHR of 1.83 (95% CI 1.57–2.14, *P* < 0.0001) for Event 1 (CVD). Notably, for CTI-CVAI, its association with the competing event (Event 2: non-CVD death) was entirely non-significant (*P* = 0.82), underscoring its highly specific predictive value for cardiovascular endpoints rather than generalized mortality (Tables S21–22). The cumulative incidence function (CIF) curves are presented in Supplementary Fig. S3.

## Discussion

In this longitudinal study based on the CHARLS cohort, we comprehensively evaluated the prognostic value of the CTI index and its seven obesity-modified derivatives for incident CVD across the CKM syndrome. Our main findings are as follows: First, all CTI-related indices maintained robust, independent positive associations with CVD risk. Notably, except for CTI-BMI, the incorporation of adiposity metrics transformed the dose-response relationship from a non-linear threshold effect to a strict monotonic linear trajectory. Second, regarding predictive performance, while the modified indices yielded non-significant increments in overall discrimination (AUC), they provided statistically significant improvements in risk reclassification (cNRI and IDI). Furthermore, these indicators demonstrate stage-dependent predictive effects, showing amplified relative risk in CKM0-2 stages, while exhibiting stronger incremental value for risk reclassification in CKM3 stage. This result suggests that the biological and clinical predictive efficacy of the predictor varies across different disease stages. Finally, mechanistic analyses revealed that central/visceral adiposity was the dominant driver of the composite risk, mediating long-term cardiovascular hazard primarily through direct metabolic-inflammatory pathways and partially through the progression of physical frailty.

The occurrence of CVDs is positively correlated with metabolic IR, while endothelial injury, dysfunction, and systemic inflammatory responses are all involved in their progression [27, 28]. Under physiological conditions, endothelial insulin signaling results in activation of endothelial nitric oxide synthase (eNOS) and endothelium-dependent, nitric oxide (NO)-mediated vasodilation, and exerts anti-inflammatory and anti-atherosclerotic effects [29, 30]. However, pathological conditions inhibit the insulin receptor substrate-1/PI3K, protein kinase B, and cyclic GMP signaling pathways, directly leading to impaired relaxation of VSMCs [31–33]. In addition, compensatory hyperinsulinemia under IR conditions excessively activates the MAPK pathway, leading to increased ET-1 production, which subsequently induces vasoconstriction and inflammatory responses, thereby exacerbating endothelial dysfunction [27]. Systemic inflammation and IR exhibit a synergistic effect, destabilizing atherosclerotic plaques [34, 35]. This instability increases the risk of plaque rupture, which may lead to thrombus formation, ultimately resulting in an increased incidence of CVDs and overall mortality [20].

The CTI has emerged as an innovative composite marker for evaluating metabolic-inflammatory dysregulation, integrating markers of IR and systemic inflammation [36]. The existing evidence comprehensively supports the predictive utility of CTI in CVD, with significant associations also reported in metabolically heterogeneous individuals and individuals with early cardiometabolic dysregulation [11, 36, 37]. However, they have not yet incorporated anthropometric indicators of obesity into the analytical models. Given the established role of excess fat, particularly visceral fat, in driving metabolic dysregulation and cardiovascular risks, combining biomarkers with anthropometric measures has become a validated strategy to enhance predictive accuracy. This method has been successfully applied to combine TyG [22] and AIP [38] with obesity indices such as BMI, WC and WHtR, demonstrating excellent potential for early identification of CVDs. Recently, the study by Zhou et al. for the first time combined CTI with obesity indicators to explore the association between CTI and stroke [36]. The improved CTI index, particularly CTI-WHtR, demonstrated a stronger association with stroke events than using CTI alone. Despite these advances, the broad associations between CTI-derived metrics and CVD remain underexplored, and their applicability and predictive power in populations with CKM syndrome are still unknown. This study fills this critical gap.

The integration of the CVAI into the CTI framework (CTI-CVAI) demonstrated a superior capacity to capture the multifaceted risk of CKM syndrome. Unlike traditional metrics like BMI, which reflect generalized adiposity and fail to distinguish between subcutaneous and visceral fat, CVAI is specifically designed to quantify visceral adipose tissue (VAT) dysfunction [39, 40]. Our findings, which highlight CTI-CVAI’s significant improvements in risk reclassification (cNRI and IDI), align with recent evidence suggesting that combining IR markers with visceral fat indices yields higher prognostic granularity for cardiometabolic outcomes [41]. A recent study by Zhang et al. similarly demonstrated that the TyG-CVAI index outperformed other obesity-modified indicators in predicting cardiometabolic multimorbidity, primarily because CVAI integrates both anthropometric and metabolic parameters to reflect the lipotoxic nature of visceral fat more effectively than BMI or WC alone [42].

The mechanistic advantage of CTI-CVAI may lie in its ability to consolidate systemic inflammation, glucose-lipid dysmetabolism, and visceral fat-related lipotoxicity. Our WQS analysis further confirms this, showing that the CVAI component carries the dominant weight within the composite model, effectively overshadowing the relative contributions of CRP or individual lipid parameters. From a pathophysiological perspective, visceral fat acts as a potent endocrine organ that secretes pro-inflammatory cytokines, directly driving the downstream inflammation (CRP) and IR (TyG) captured by the CTI index [43, 44]. By incorporating CVAI, the CTI-CVAI index provides a more direct assessment of the “upstream” driver of cardiovascular damage. However, the marginal increment in discriminative ability observed in the study results suggests that the clinical benefits of this increased computational complexity (incorporating multiple biochemical and anthropometric inputs) remain debatable, especially in real-world settings.

This national prospective cohort study possesses several notable methodological and clinical strengths. First, it provides the first comprehensive longitudinal evidence evaluating the CTI framework and its obesity-modified derivatives across the CKM syndrome continuum. By rigorously incorporating the Fine-Gray competing risk model, we ensured that the robust associations observed between these indices (particularly CTI-CVAI) and incident CVD are truly independent of the high mortality risk inherent in an aging population. Second, our predictive evaluation methodology is exceptionally rigorous; rather than relying on traditional, potentially biased comparative tests, we employed time-dependent NNE with Bootstrap resampling for AUC comparisons, alongside cNRI and IDI, to assess risk stratification from multiple dimensions accurately. Third, by capturing cumulative exposure and mapping dynamic longitudinal trajectories, this study accounts for the temporal accumulation of metabolic-inflammatory burden and adiposity, overcoming the critical limitations of single-timepoint baseline analyses. Finally, the innovative integration of WQS regression and causal mediation analysis provides profound mechanistic insights. This approach not only quantified the dominant pathophysiological weight of visceral fat but also uniquely identified physical frailty as a crucial mediator, effectively bridging the gap between statistical prediction and clinical etiology.

Several limitations of the present study must be acknowledged. First, regarding the operationalization of the CKM stages, our study was constrained by the inherent data limitations of the CHARLS cohort. Specifically, comprehensive quantification of the Urine Albumin-Creatinine Ratio (UACR) was unavailable for the full baseline cohort, meaning renal impairment in CKM Stage 2 was defined primarily based on eGFR and physician diagnosis. The absence of microalbuminuria data may lead to the under-detection of early kidney damage. Furthermore, the accurate classification of CKM Stage 3 was also limited. According to the 2023 AHA guidelines, Stage 3 is characterized by subclinical CVD, typically diagnosed via advanced imaging (e.g., coronary artery calcium scoring, echocardiography) or specific cardiac biomarkers (e.g., NT-proBNP, hs-cTn). The lack of these subclinical assessments in this epidemiological survey inevitably leads to the under-ascertainment of Stage 3 cases, potentially misclassifying some individuals with undetected subclinical target organ damage into Stage 2. Although this non-differential misclassification may blur the strict boundaries between intermediate stages, the overarching prognostic trajectories of the composite indices across the CKM continuum remained remarkably robust. Second, the study population was restricted to middle-aged and older Chinese adults, which may limit the generalizability of our findings to younger populations or different ethnic groups. Third, although we utilized a validated time-weighted average approach to calculate cumulative exposure, the CHARLS database only collected blood samples during two specific waves (2011/2012 and 2015). This infrequent sampling restricts a more granular assessment of short-term dynamic fluctuations in CTI-related indices. Finally, despite rigorous adjustment for a comprehensive set of covariates, residual confounding from unmeasured or uncontrolled factors—such as specific medication adherence (e.g., lipid-lowering or anti-diabetic drugs) and detailed dietary habits—cannot be entirely ruled out.

## Conclusions

In conclusion, this study demonstrates that integrating obesity-related metrics, most notably the CVAI, into the CTI framework provides a more refined assessment of cardiovascular risk across the CKM syndrome. While they provide only marginal and non-significant increments in overall discriminative ability (AUC), these obesity-modified indices successfully transform the dose-response relationship into a monotonic linear risk trajectory and significantly enhance risk reclassification (cNRI and IDI). Furthermore, the CKM hierarchical analysis indicates that the incremental utility of these complex composite indicators is stage-dependent, with amplified relative risks in CKM early to mid-stage and stronger incremental value for risk reclassification in the CKM late stage. Mechanistically, visceral adiposity serves as the primary driver of this composite risk, operating partly through the progression of physical frailty. Ultimately, the trade-off between increased computational complexity and limited predictive gain highlights the need for a balanced clinical approach, favoring sophisticated metrics like CTI-CVAI for targeted risk refinement while retaining simpler tools for broad-based population screening.

## Supplementary Information

Below is the link to the electronic supplementary material.

Supplementary Material 1.

## Data Availability

The dataset supporting the conclusions of this article is available in the CHARLS dataset(http://charls.pku.edu.cn/en).

## Abbreviations

CVD: Cardiovascular disease
CKD: Chronic kidney disease
CKM: Cardiovascular-kidney-metabolic
CTI: C-reactive protein-triglyceride glucose index
IR: Insulin resistance
BMI: Body Mass Index
CHARLS: China Health and Retirement Longitudinal Study
CRP: C-reactive protein
TG: Triglycerides
FBG: Fasting blood glucose
WC: Waist circumference
SBP: Systolic blood pressure
DBP: Diastolic blood pressure
HbA1c: Glycated hemoglobin A1c
TC: Total cholesterol
HDL-C: High-density lipoprotein cholesterol
LDL-C: Low-density lipoprotein cholesterol
Scr: Serum creatinine
BUN: Blood urea nitrogen
UA: Uric acid
eGFR: Estimated glomerular filtration rate
CIF: Cumulative incidence function
VIF: Variance inflation factor
NNE: Nearest Neighbor Estimation
SD: Standard deviation
RCS: Restricted cubic spline
ROC: Receiver operating characteristic
AUC: Area under the curve
cNRI: Continuous Net Reclassification Improvement
IDI: Integrated Discrimination Improvement
WQS: Weighted quantile sum
FI: Frailty index
IQR: Interquartile range
eNOS: Endothelial nitric oxide synthase
NO: Nitric oxide
VAT: Visceral adipose tissue
