# Supplementary material for: Association of cumulative exposure and dynamic trajectories of the C-reactive protein-triglyceride-glucose and its modified indices with cardiovascular disease in individuals with cardiovascular-kidney-metabolic syndrome stages 0–3: a longitudinal analysis based on CHARLS
Source: Cardiovasc Diabetol. 2026 May 11;25:192. doi: 10.1186/s12933-026-03197-x (PMC13330237; doi:10.1186/s12933-026-03197-x)
Supplement: Supplementary file 1 — Supplementary Material 1. [file 12933_2026_3197_MOESM1_ESM.docx]

**Figure S1.** Clustering of control trajectories in cumulative CTI and its modified indices from 2011 to 2015.

**Figure S2.** Mediation analysis of HbA1c in the association between CTI and its modified indices and new-onset CVD.

**Figure S2.** Cumulative incidence function (CIF) curves and subdistribution hazard ratios (subHR) for cardiovascular disease (CVD) and non-CVD death across quartiles of different CTI-related indices.

**TableS1.** Distribution of variables with missing data

**Table S2.** Methods for evaluating CKM syndrome stage 0-4

**Table S3.** Specific definitions of various diseases

**Table S4.** Variance Inflation Factor test results of independent variables in different CTI and its modified indices Models

**Table S5.** Variance Inflation Factor test results of independent variables in different cumulative CTI and its modified indices Models

**Table S6.** Proportional Hazards hypothesis test results for CTI and its modified indices.

**Table S7.** Proportional Hazards hypothesis test results for cumulative CTI and its modified indices.

**Table S8** The detailed list of health deficits items included in the FI.

**Table S9.** The baseline characteristics stratified by CKM syndrome stage 0-3

**Table S10.** Associations between CTI and its related indices and CVD risk in CKM syndrome stage 0-2 stage

**Table S11.** Associations between CTI and its related indices and CVD risk in CKM syndrome stage 3

**Table S12.** Threshold effect analysis of CTI on CVD risk using a two-piecewise linear regression model

**Table S13.** Threshold effect analysis of CTI-BMI on CVD risk using a two-piecewise linear regression model

**Table S14.** ROC curve analysis of CTI and related indices.

**Table S15.** NRI and IDI curve analysis of CTI and related indices.

**Table S16.** ROC curve analysis of cumulative CTI and related indices.

**Table S17.** NRI and IDI curve analysis of cumulative CTI and related indices.

**Table S18.** Baseline characteristics comparison between excluded and included participants

**Table S19.** Associations between CTI and its related indices(per IQR) and CVD risk in CKM syndrome stage 0-3

**Table S20.** Associations between Cumulative CTI and its related indices(per IQR) and CVD risk in CKM syndrome stage 0-3

**Table S21.** Gray’s Test for Cumulative Incidence of CVD and Non-CVD Death by CTI Quartiles

**Table S22.** Association of CTI and its related indices and CVD risk in CKM syndrome stage 0-3 in the Competing Risks Model

**Figure S1.** Clustering of control trajectories in cumulative CTI and its modified indices from 2011 to 2015. Three clusters were identified using the k-means method with Euclidean distance. Each cluster is represented by a unique color and shape across all figures to distinguish between groups and highlight variations over time.**
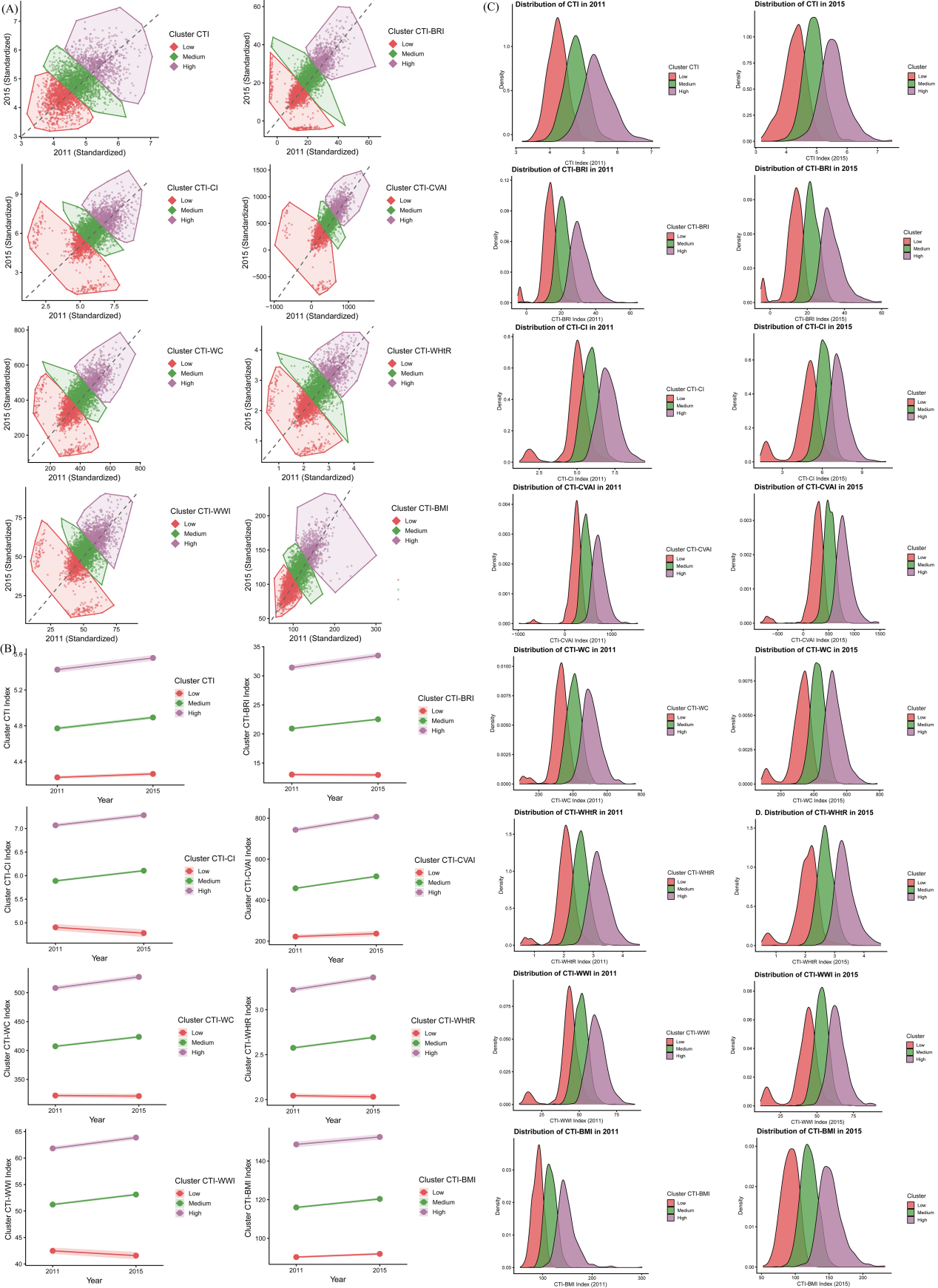
**

**Figure S2.** Mediation analysis of HbA1c in the association between CTI and its modified indices and new-onset CVD.

**
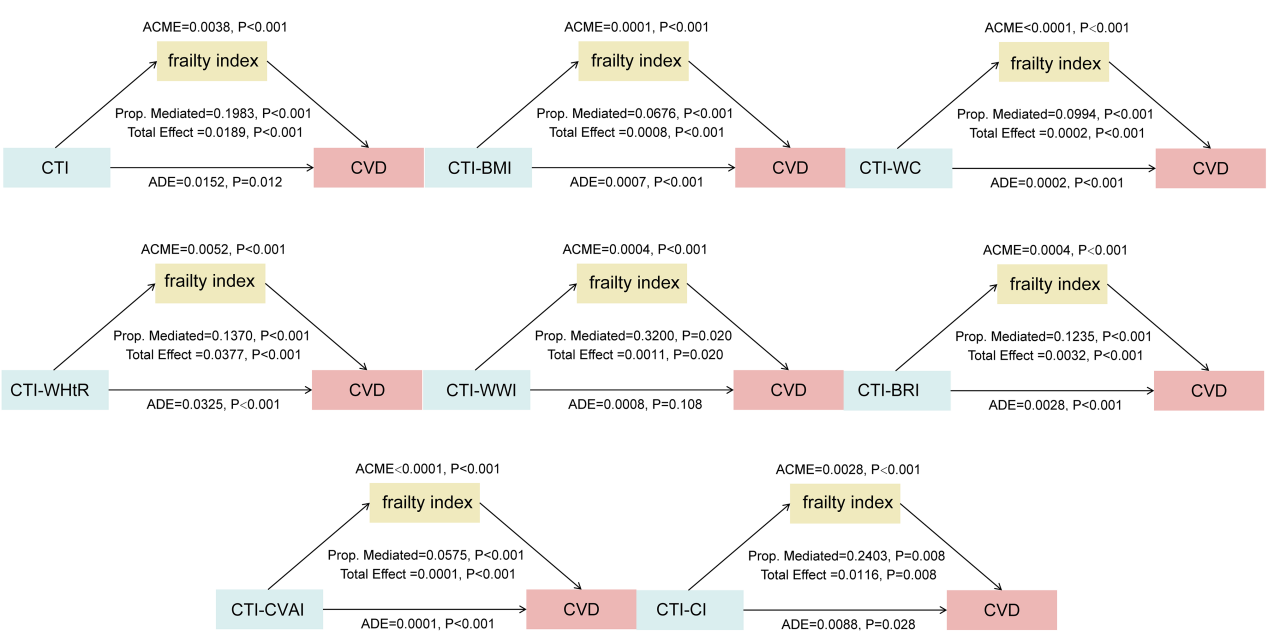
**

**Figure S3.** Cumulative incidence function (CIF) curves and subdistribution hazard ratios (subHR) for cardiovascular disease (CVD) and non-CVD death across quartiles of different CTI-related indices. The left panels show the forest plots of subHRs (95% CI) derived from multivariable Fine-Gray competing risk models, with blue dots representing CVD events and red dots representing non-CVD death. The right panels display the CIF curves for CVD events, stratified by CTI quartiles (Q1: lowest, Q4: highest). Gray’s test was used to compare the differences in cumulative incidence across quartiles. All models were adjusted for age, sex, marital status, education, eGFR, smoke status, drink status, residence place, BUN, UA, LDL-C, HDL-C and HbA1c.

**
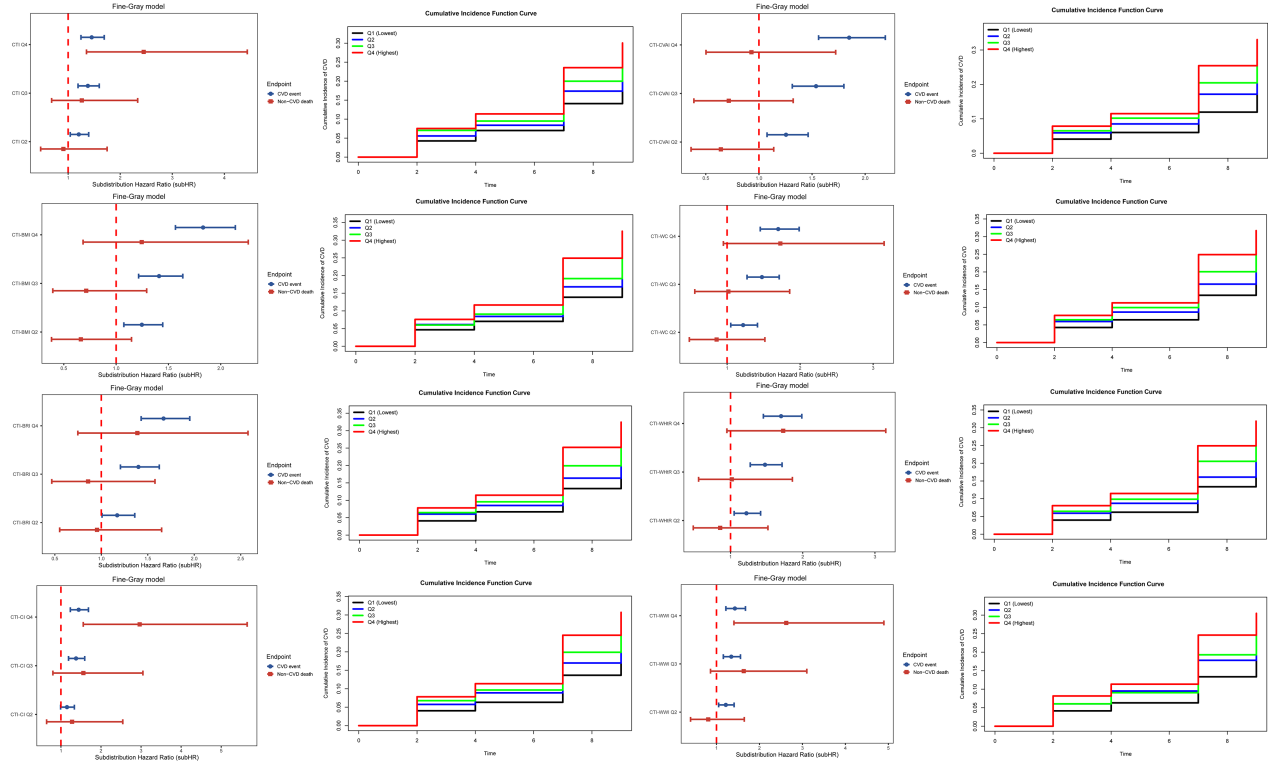
**

**TableS1.** Distribution of variables with missing data

| Variables | Number of Missing | Proporation(%) |
| --- | --- | --- |
| HbA1c | 101 | 0.86 |
| BUN | 118 | 1.00 |
| UA | 117 | 1.00 |
| HDL-C | 118 | 1.00 |
| LDL-C | 133 | 1.13 |
| eGFR | 138 | 1.17 |
| Education | 9 | 0.08 |
| Smoke | 3 | 0.03 |
| Drink | 4 | 0.03 |
| CRP | 117 | 1.29 |
| TG | 123 | 0.99 |
| FBG | 1046 | 8.86 |
| WC | 1305 | 11.05 |
| height | 1335 | 11.31 |
| weight | 1309 | 11.09 |

HbA1c: Glycosylated Hemoglobin, Type A1C; BUN: blood urea nitrogen; UA: uric acid; HDL-C: high density lipoprotein cholesterol; LDL-C: low density lipoprotein cholesterol; eGFR: estimated glomerular filtration rate; CRP: C-reactive protein; TG: triglyceride; FBG: fasting blood glucose; WC:waist circumference.

**Table S2.** Methods for evaluating CKM syndrome stage 0-4

| CKM stages | Threshold for CKM conditions |
| --- | --- |
| Stage 0: No CKM risk factors | All criteria are met：  ①BMI< 23 kg/m2;  ②Waist circumference <80/90 cm in female/male;  ③Fasting blood glucose < 100 mg/dL and HbA1c < 5.7% and without self-reported diagnosis of diabetes;  ④SBP <130 mm Hg and DBP <80 mm Hg without self-reported diagnosis of hypertension or use of antihypertensive medications;  ⑤HDL-C <50/40 mg/dL in female/male;  ⑥TG < 150 mg/dL;  ⑦eGFR ≥ 60 ml/min/1.73m2 and without self-reported diagnosis of CKD.  No Subclinical CVD and clinical CVD. |
| Stage 1: Excess or dysfunctional adiposity | Any of the three criteria is met：  ①BMI ≥23 kg/m2;  ②Waist circumference ≥80/90 cm in female/male;  ③Fasting blood glucose ≥100 mg/dL or HbA1c between 5.7% and 6.4%;  All criteria are met：  ①SBP <130 mmHg and DBP <80 mmHg without self-reported diagnosis of hypertension;  ②HDL-C <50/40 mg/dL in female/male;  ③TG < 150 mg/dL;  ④eGFR ≥ 60 ml/min/1.73m2 and without self-reported diagnosis of CKD;  ⑤No Subclinical CVD and clinical CVD. |
| Stage 2: Metabolic risk factors and CKD | Any of the five criteria is met:  ①TG≥135 mg/dL;  ②Hypertension;  ③Diabetes;  ④Metabolic syndrome;  ⑤eGFR：30-60 ml/min/1.73m2 and/or with self-reported diagnosis of CKD;  All criteria are met：  No Subclinical CVD and clinical CVD. |
| Stage 3: Subclinical CVD in CKM | Any of the two criteria is met:  ①eGFR<30 ml/min/1.73m2;  ②High predicted 10-y CVD risk;  The criterion is met：  ①No clinical CVD. |
| Stage 4: Clinical CVD | Clinical CVD (including self-reported diagnosed CVD, including heart failure, coronary heart disease, angina, heart attack, and stroke) |

BMI: body mass index; SBP: Systolic blood pressure; DBP: Diastolic blood pressure; HbA1c: Glycosylated Hemoglobin, Type A1C; HDL-C: high density lipoprotein cholesterol; TG: total cholesterol; LDL-C: low density lipoprotein cholesterol; Scr: serum creatinine; BUN: blood urea nitrogen; eGFR: estimated glomerular filtration rate; CKM: cardiovascular–kidney–metabolic; CVD: cardiovascular disease; CKD: chronic kidney disease.

**Table S3.** Specific definitions of various diseases

| Disease name | Definition of Disease |
| --- | --- |
| Dyslipidemia | Any of the three criteria is met：  ①TG ≥150 mg/dL;  ②TC ≥ 6.2mmol/L;  ③LDL-C ≥ 4.1mmol/L; |
| Hypertension | Any of the three criteria is met：  ①SBP ≥130 mm Hg or DBP ≥80 mm Hg;  ②Self-reported diagnosis of hypertension;  ③Use of antihypertensive medications. |
| Diabetes | Any of the four criteria is met：  ①FBG ≥7mmol/l;  ②Random blood glucose ≥11.1mmol/l;  ③HbA1c ≥6.5%;  ④Self-reported diagnosis of diabetes mellitus. |
| Metabolic Syndrome | Meet any three or more of the five：  ①Waist circumference ≥85/90 cm in female/male;  ②Fasting blood glucose ≥100 mg/dL or oral glucose tolerance test (OGTT) 2-hour blood glucose ≥7.8 mmol/L or self-reported diagnosis of diabetes, use of insulin, or oral hypoglycemic agents;  ③SBP >130 mm Hg and DBP >85 mm Hg or self-reported diagnosis of hypertension or use of antihypertensive medications;  ④TG ≥150 mg/dL;  ⑤HDL-C ＜50/40 mg/dL in female/male. |

HbA1c: Glycosylated Hemoglobin, Type A1C; FBG: fast blood glucose; HDL-C: high density lipoprotein cholesterol; TG: total cholesterol; TC: total cholesterol; LDL-C: low density lipoprotein cholesterol; OGTT: oral glucose tolerance test

**Table S4.** Variance Inflation Factor test results of independent variables in different CTI and its modified indices Models

|  | CTI | CTI-BMI | CTI-WC | CTI-WHtR | CTI-WWI | CTI-BRI | CTI-CVAI | CTI-CI |
| --- | --- | --- | --- | --- | --- | --- | --- | --- |
| index | 1.395998 | 1.391556 | 1.440998 | 1.440998 | 1.406329 | 1.409720 | 1.702189 | 1.368444 |
| age | 1.652416 | 1.669967 | 1.654032 | 1.654032 | 1.715792 | 1.686288 | 1.739252 | 1.695487 |
| Sex(male) | 2.625196 | 2.652345 | 2.640944 | 2.640944 | 2.713855 | 2.775846 | 2.646953 | 2.657087 |
| Marital status(Other) | 1.126108 | 1.129885 | 1.126934 | 1.126934 | 1.126543 | 1.126268 | 1.130571 | 1.126599 |
| Education(illiterate) | 3.412739 | 3.428999 | 3.422613 | 3.422613 | 3.420642 | 3.428934 | 3.436656 | 3.417722 |
| Education (Junior high school and below) | 2.974382 | 2.972599 | 2.972606 | 2.972606 | 2.977927 | 2.977264 | 2.970642 | 2.975813 |
| eGFR | 1.753075 | 1.762473 | 1.749903 | 1.749903 | 1.754892 | 1.760392 | 1.758228 | 1.752941 |
| Smoke(Ever) | 1.248395 | 1.254585 | 1.250439 | 1.250439 | 1.248353 | 1.250862 | 1.254970 | 1.248325 |
| Smoke(Never) | 2.293895 | 2.294847 | 2.300249 | 2.300249 | 2.295384 | 2.303406 | 2.289352 | 2.296341 |
| Drink(yes) | 1.361374 | 1.366626 | 1.370737 | 1.370737 | 1.360230 | 1.365564 | 1.372952 | 1.361869 |
| Residence place(urban) | 1.094449 | 1.101265 | 1.092939 | 1.092939 | 1.092535 | 1.091095 | 1.095012 | 1.092168 |
| BUN | 1.152515 | 1.149853 | 1.149181 | 1.149181 | 1.149698 | 1.147976 | 1.145505 | 1.149530 |
| UA | 1.530300 | 1.531859 | 1.546317 | 1.546317 | 1.531306 | 1.533212 | 1.550973 | 1.531078 |
| LDL-C | 1.059521 | 1.063902 | 1.064569 | 1.064569 | 1.060298 | 1.067053 | 1.063912 | 1.060285 |
| HDL-C | 1.285771 | 1.289827 | 1.328118 | 1.328118 | 1.235859 | 1.236041 | 1.523354 | 1.252692 |
| HbA1c | 1.135869 | 1.085858 | 1.114775 | 1.114775 | 1.110866 | 1.082695 | 1.091851 | 1.112073 |

**Table S5.** Variance Inflation Factor test results of independent variables in different cumulative CTI and its modified indices Models

|  | CTI | CTI-BMI | CTI-WC | CTI-WHtR | CTI-WWI | CTI-BRI | CTI-CVAI | CTI-CI |
| --- | --- | --- | --- | --- | --- | --- | --- | --- |
| Cumulative index | 1.422901 | 1.441712 | 1.447172 | 1.512921 | 1.486388 | 1.443568 | 1.569683 | 1.430078 |
| age | 1.629657 | 1.661506 | 1.639458 | 1.645025 | 1.673264 | 1.654837 | 1.679571 | 1.657500 |
| Sex(male) | 2.691698 | 2.714413 | 2.685161 | 2.812060 | 2.808652 | 2.842431 | 2.696191 | 2.726242 |
| Marital status(Other) | 1.118767 | 1.119947 | 1.120547 | 1.120810 | 1.120930 | 1.122969 | 1.124092 | 1.120463 |
| Education(illiterate) | 3.839712 | 3.855818 | 3.849695 | 3.847304 | 3.839573 | 3.852262 | 3.866485 | 3.838306 |
| Education (Junior high school and below) | 3.382841 | 3.376201 | 3.373219 | 3.375226 | 3.376959 | 3.374470 | 3.374991 | 3.374960 |
| eGFR | 1.703600 | 1.706564 | 1.705778 | 1.704922 | 1.707330 | 1.710460 | 1.709846 | 1.707135 |
| Smoke(Ever) | 1.210066 | 1.212374 | 1.212262 | 1.704922 | 1.210044 | 1.213756 | 1.216090 | 1.210080 |
| Smoke(Never) | 2.243681 | 2.234730 | 2.239162 | 2.243080 | 2.241396 | 2.241752 | 2.226435 | 2.241604 |
| Drink(yes) | 1.403845 | 1.403906 | 1.413401 | 1.413231 | 1.407438 | 1.411482 | 1.405962 | 1.408283 |
| Residence place(urban) | 1.080882 | 1.076930 | 1.075461 | 1.075614 | 1.077048 | 1.073402 | 1.075260 | 1.077035 |
| BUN | 1.145592 | 1.149646 | 1.147815 | 1.148981 | 1.148439 | 1.150001 | 1.144272 | 1.147422 |
| UA | 1.529541 | 1.542787 | 1.545884 | 1.547561 | 1.533219 | 1.534712 | 1.543612 | 1.533585 |
| LDL-C | 1.084578 | 1.093627 | 1.089770 | 1.092048 | 1.085103 | 1.091713 | 1.087726 | 1.084719 |
| HDL-C | 1.316309 | 1.319945 | 1.348131 | 1.320256 | 1.280787 | 1.253178 | 1.461978 | 1.301130 |
| HbA1c | 1.142678 | 1.092981 | 1.108210 | 1.108365 | 1.114580 | 1.078614 | 1.080979 | 1.116436 |

**Table S6.** Proportional Hazards hypothesis test results for CTI and its modified indices.

| Model | Overall_p | CKM0_p | CKM1_p | CKM2_p | CKM3_p |
| --- | --- | --- | --- | --- | --- |
| CTI | 0.555 | 0.617 | 0.644 | 0.618 | 0.875 |
| CTI_Q | 0.618 | 0.667 | 0.405 | 0.553 | 0.842 |
| CTI-BMI | 0.607 | 0.602 | 0.691 | 0.646 | 0.862 |
| CTI-BMI_Q | 0.637 | 0.704 | 0.732 | 0.749 | 0.769 |
| CTI-WC | 0.474 | 0.547 | 0.698 | 0.602 | 0.849 |
| CTI-WC_Q | 0.642 | 0.657 | 0.392 | 0.304 | 0.87 |
| CTI-WHtR | 0.474 | 0.547 | 0.698 | 0.602 | 0.849 |
| CTI-WHtR_Q | 0.631 | 0.529 | 0.635 | 0.564 | 0.735 |
| CTI-WWI | 0.362 | 0.455 | 0.705 | 0.541 | 0.825 |
| CTI-WWI_Q | 0.1 | 0.553 | 0.801 | 0.537 | 0.166 |
| CTI-BRI | 0.523 | 0.438 | 0.651 | 0.594 | 0.866 |
| CTI-BRI_Q | 0.529 | 0.696 | 0.753 | 0.558 | 0.876 |
| CTI-CVAI | 0.518 | 0.556 | 0.696 | 0.623 | 0.871 |
| CTI-CVAI_Q | 0.445 | NA | 0.741 | 0.703 | 0.559 |
| CTI-CI | 0.36 | 0.476 | 0.698 | 0.585 | 0.81 |
| CTI-CI_Q | 0.407 | 0.62 | 0.681 | 0.519 | 0.494 |

**Table S7.** Proportional Hazards hypothesis test results for cumulative CTI and its modified indices.

| Model | Overall_p | CKM0_p | CKM1_p | CKM2_p | CKM3_p |
| --- | --- | --- | --- | --- | --- |
| Cumulative CTI | 0.898 | 0.881 | 0.543 | 0.634 | 0.708 |
| Cumulative CTI_Q | 0.852 | 0.861 | 0.6 | 0.605 | 0.747 |
| Cumulative CTI-BMI | 0.915 | 0.868 | 0.52 | 0.632 | 0.654 |
| Cumulative CTI-BMI_Q | 0.94 | NA | 0.618 | 0.764 | 0.539 |
| Cumulative CTI-WC | 0.868 | 0.881 | 0.531 | 0.601 | 0.709 |
| Cumulative CTI-WC_Q | 0.952 | 0.809 | 0.635 | 0.758 | 0.8 |
| Cumulative CTI-WHtR | 0.877 | 0.88 | 0.545 | 0.591 | 0.71 |
| Cumulative CTI-WHtR_Q | 0.934 | NA | 0.379 | 0.757 | 0.36 |
| Cumulative CTI-WWI | 0.84 | 0.882 | 0.52 | 0.614 | 0.703 |
| Cumulative CTI-WWI_Q | 0.85 | 0.882 | 0.672 | 0.496 | 0.628 |
| Cumulative CTI-BRI | 0.88 | 0.858 | 0.551 | 0.597 | 0.713 |
| Cumulative CTI-BRI_Q | 0.923 | NA | 0.6 | 0.767 | 0.65 |
| Cumulative CTI-CVAI | 0.902 | 0.875 | 0.543 | 0.639 | 0.692 |
| Cumulative CTI-CVAI_Q | 0.873 | NA | 0.567 | 0.728 | 0.592 |
| Cumulative CTI-CI | 0.833 | 0.881 | 0.51 | 0.616 | 0.701 |
| Cumulative CTI-CI_Q | 0.886 | 0.84 | 0.627 | 0.714 | 0.587 |

**Table S8** The detailed list of health deficits items included in the FI.

| No | Description of the items | Cut-off value |
| --- | --- | --- |
|  | CHARLS |  |
| 1 | Self-reported physician diagnosed hypertension | Yes = 1, No = 0 |
| 2 | Self-reported physician diagnosed diabetes | Yes = 1, No = 0 |
| 3 | Self-reported physician diagnosed heart disease | Yes = 1, No = 0 |
| 4 | Self-reported physician diagnosed stroke | Yes = 1, No = 0 |
| 5 | Self-reported physician diagnosed cancer | Yes = 1, No = 0 |
| 6 | Self-reported physician diagnosed arthritis | Yes = 1, No = 0 |
| 7 | Self-reported physician diagnosed chronic lung disease | Yes = 1, No = 0 |
| 8 | Self-reported physician diagnosed asthma | Yes = 1, No = 0 |
| 9 | Self-reported physician diagnosed any emotional, nervous, or psychiatric problems | Yes = 1, No = 0 |
| 10 | Self-reported physician diagnosed memory-related disease | Yes = 1, No = 0 |
| 11 | Self-reported vision problems | Yes = 1, No = 0 in the CHARLS |
| 12 | Self-reported hearing problems | Yes = 1, No = 0 in the CHARLS |
| 13 | Self-reported general health status | Poor or fair = 1, excellent, very good, or good = 0 |
| 14 | Difficulty with dressing | Yes = 1, No = 0 |
| 15 | Difficulty with bathing or showering | Yes = 1, No = 0 |
| 16 | Difficulty with eating | Yes = 1, No = 0 |
| 17 | Difficulty with getting in and out of bed | Yes = 1, No = 0 |
| 18 | Difficulty with using the toilet | Yes = 1, No = 0 |
| 19 | Difficulty with managing money | Yes = 1, No = 0 |
| 20 | Difficulty with taking medications | Yes = 1, No = 0 |
| 21 | Difficulty with shopping for groceries | Yes = 1, No = 0 |
| 22 | Difficulty with preparing meals | Yes = 1, No = 0 |
| 23 | Difficulty with doing housework | Yes = 1, No = 0 |
| 24 | Mobility: difficulty with walking 100 yards | Yes = 1, No = 0 |
| 25 | Mobility: difficulty with getting up from a chair after sitting for long periods | Yes = 1, No = 0 |
| 26 | Mobility: difficulty with climbing several flights of stairs without resting | Yes = 1, No = 0 |
| 27 | Mobility: difficulty with lifting or carrying weights over 10 pounds/jins | Yes = 1, No = 0 |
| 28 | Mobility: difficulty with picking up a coin from the table | Yes = 1, No = 0 |
| 29 | Mobility: difficulty with stooping, kneeling, or crouching | Yes = 1, No = 0 |
| 30 | Mobility: difficulty with reaching arms above shoulder level | Yes = 1, No = 0 |
| 31 | Depression: CESD-10 questionnaire | CESD-10 >10 =1, ≤10 =0 in 32the CHARLS |
| 32 | Cognition: (memory test score + orientation test score) / 14 | Continuous, ranging from 0 to 1 |

Heart disease indicates the angina, coronary heart disease, congestive heart failure, or other heart problems.

Memory-related disease indicates Alzheimer’s disease or dementia, organic brain senility, or other serious memory impairment.

Depression is evaluated using Center for Epidemiologic Studies Depression Scale (CESD). In the CHARLS, CESD-10 is used, and the total score ranges from 0 to 30. The higher score indicates more severe depressive symptoms.

The memory score is the average of words that are not recalled in the immediate and delayed word recall tasks. The memory score ranges from 0 to 10. The orientation test comprises four questions about the day of the week, the month, the date of the month, and the year. One point is given for each wrong answer, and the range is from 0 to 4.

**Table S9.** The baseline characteristics stratified by CKM syndrome stage 0-3

| Variable | Total (n=7,118) | CKM0 stage (n=560) | CKM1 stage (n=1,307) | CKM2 stage (n=2,736) | CKM3 stage (n=2,515) | *p* value |
| --- | --- | --- | --- | --- | --- | --- |
| Sex, n (%) |  |  |  |  |  | <0.001 |
| female | 3850(54.09) | 239(42.68) | 806(61.67) | 1876(68.57) | 929(36.94) |  |
| male | 3268(45.91) | 321(57.32) | 501(38.33) | 860(31.43) | 1586(63.06) |  |
| Age, years | 58.52 ± 9.48 | 56.01 ± 8.14 | 54.82 ± 7.72 | 54.96 ± 7.94 | 64.86 ± 8.81 | <0.001 |
| Educational level, n (%) |  |  |  |  |  | <0.001 |
| above junior high school | 707( 9.93) | 68(12.14) | 121( 9.26) | 313(11.44) | 205( 8.15) |  |
| illiterate | 3383(47.53) | 260(46.43) | 592(45.29) | 1225(44.77) | 1306(51.93) |  |
| junior high school and below | 3028(42.54) | 232(41.43) | 594(45.45) | 1198(43.79) | 1004(39.92) |  |
| Marital status,  n (%) |  |  |  |  |  | <0.001 |
| married | 6321(88.80) | 521(93.04) | 1209(92.50) | 2478(90.57) | 2113(84.02) |  |
| other | 797(11.20) | 39( 6.96) | 98( 7.50) | 258( 9.43) | 402(15.98) |  |
| Residence place,  n (%) |  |  |  |  |  | <0.001 |
| rural | 4672(65.64) | 407(72.68) | 897(68.63) | 1755(64.14) | 1613(64.14) |  |
| urban | 2446(34.36) | 153(27.32) | 410(31.37) | 981(35.86) | 902(35.86) |  |
| Smoke, n (%) |  |  |  |  |  | <0.001 |
| current | 2140(30.06) | 200(35.71) | 277(21.19) | 391(14.29) | 1272(50.58) |  |
| ever | 578( 8.12) | 49( 8.75) | 88( 6.73) | 211( 7.71) | 230( 9.15) |  |
| never | 4400(61.82) | 311(55.54) | 942(72.07) | 2134(78.00) | 1013(40.28) |  |
| Drink, n (%) |  |  |  |  |  | <0.001 |
| no | 4701(66.04) | 341(60.89) | 902(69.01) | 1965(71.82) | 1493(59.36) |  |
| yes | 2417(33.96) | 219(39.11) | 405(30.99) | 771(28.18) | 1022(40.64) |  |
| Hypertension,  n (%) |  |  |  |  |  | <0.001 |
| no | 3287(46.18) | 560(100.00) | 1307(100.00) | 783(28.62) | 637(25.33) |  |
| yes | 3831(53.82) |  |  | 1953(71.38) | 1878(74.67) |  |
| Diabetes, n (%) |  |  |  |  |  | <0.001 |
| no | 6090(85.56) | 560(100.00) | 1307(100.00) | 2511(91.78) | 1712(68.07) |  |
| yes | 1028(14.44) |  |  | 225( 8.22) | 803(31.93) |  |
| Dyslipidemian,  n (%) |  |  |  |  |  | <0.001 |
| no | 4418(62.07) | 518(92.50) | 1068(81.71) | 1676(61.26) | 1156(45.96) |  |
| yes | 2700(37.93) | 42( 7.50) | 239(18.29) | 1060(38.74) | 1359(54.04) |  |
| BMI(kg/m2) | 23.48 ± 3.81 | 20.10 ± 1.72 | 23.15 ± 3.23 | 24.35 ± 3.88 | 23.46 ± 3.89 | <0.001 |
| WC(cm) | 84.09 ± 12.15 | 74.35 ± 8.51 | 82.16 ± 10.80 | 85.55 ± 11.86 | 85.67 ± 12.66 | <0.001 |
| SBP(mmHg) | 129.63 ± 20.99 | 111.00 ± 9.89 | 113.61 ± 8.85 | 132.40 ± 19.98 | 139.06 ± 21.02 | <0.001 |
| DBP(mmHg) | 75.53 ± 12.00 | 65.52 ± 7.46 | 67.58 ± 7.07 | 78.81 ± 11.85 | 78.33 ± 11.92 | <0.001 |
| HbA1c(%) | 5.27 ± 0.79 | 5.00 ± 0.35 | 5.13 ± 0.38 | 5.17 ± 0.59 | 5.50 ± 1.10 | <0.001 |
| HDL-C(mg/dL) | 51.90 ± 15.18 | 60.87 ± 12.23 | 57.81 ± 14.07 | 50.84 ± 14.34 | 47.99 ± 15.48 | <0.001 |
| LDL-C(mg/dL) | 3.04 ± 0.90 | 2.82 ± 0.74 | 2.99 ± 0.77 | 2.96 ± 0.87 | 3.19 ± 1.01 | <0.001 |
| Scr(mg/dL) | 0.78 ± 0.24 | 0.77 ± 0.16 | 0.73 ± 0.15 | 0.74 ± 0.17 | 0.84 ± 0.32 | <0.001 |
| BUN(mg/dL) | 15.66 ± 4.52 | 15.84 ± 4.60 | 15.46 ± 4.13 | 15.11 ± 4.29 | 16.34 ± 4.84 | <0.001 |
| UA(mg/dL) | 4.41 ± 1.23 | 4.18 ± 1.06 | 4.06 ± 1.03 | 4.30 ± 1.18 | 4.76 ± 1.32 | <0.001 |
| FBG(mg/dL) | 108.89 ± 33.32 | 90.70 ± 6.97 | 100.89 ± 10.05 | 105.07 ± 21.86 | 121.25 ± 47.79 | <0.001 |
| TG(mg/dL) | 127.07 ± 91.37 | 76.03 ± 23.89 | 82.29 ± 24.32 | 135.65 ± 83.30 | 152.38 ± 115.36 | <0.001 |
| CRP(mg/dL) | 2.63 ± 7.42 | 2.15 ± 6.16 | 2.13 ± 7.13 | 2.35 ± 6.15 | 3.31 ± 8.89 | <0.001 |
| eGFR(ml/min·1.73m²) | 96.49 ± 14.01 | 100.17 ± 10.69 | 101.16 ± 11.09 | 98.86 ± 13.29 | 90.66 ± 14.81 | <0.001 |
| CVD, n (%) |  |  |  |  |  | <0.001 |
| No | 5501(77.28) | 488(87.14) | 1070(81.87) | 2111(77.16) | 1832(72.84) |  |
| Yes | 1617(22.72) | 72(12.86) | 237(18.13) | 625(22.84) | 683(27.16) |  |
| CTI | 4.73 ± 0.57 | 4.33 ± 0.47 | 4.45 ± 0.46 | 4.74 ± 0.52 | 4.95 ± 0.60 | <0.001 |
| CTI-BMI | 111.63 ± 25.55 | 87.04 ± 11.44 | 103.07 ± 18.00 | 116.05 ± 25.21 | 116.73 ± 27.18 | <0.001 |
| CTI-WC | 399.44 ± 84.77 | 322.37 ± 52.18 | 365.91 ± 62.65 | 407.07 ± 79.12 | 425.72 ± 90.70 | <0.001 |
| CTI-WHtR | 2.53 ± 0.54 | 2.03 ± 0.32 | 2.33 ± 0.41 | 2.60 ± 0.51 | 2.69 ± 0.59 | <0.001 |
| CTI-WWI | 52.31 ± 9.42 | 45.43 ± 7.06 | 48.50 ± 7.66 | 52.62 ± 8.53 | 55.49 ± 10.11 | <0.001 |
| CTI-BRI | 19.77 ± 8.12 | 12.36 ± 3.65 | 17.36 ± 6.00 | 20.87 ± 7.79 | 21.48 ± 8.90 | <0.001 |
| CTI-CVAI | 444.97 ± 241.23 | 214.32 ± 131.79 | 338.17 ± 164.97 | 460.75 ± 211.58 | 534.65 ± 268.21 | <0.001 |
| CTI-CI | 6.02 ± 1.06 | 5.25 ± 0.81 | 5.58 ± 0.86 | 6.04 ± 0.96 | 6.40 ± 1.13 | <0.001 |

Continuous variables are expressed as Mean ± SD or Median (IQR), categorical variables are expressed as number (percent). BMI: body mass index; SBP: Systolic blood pressure; DBP: Diastolic blood pressure; HbA1c: Glycosylated Hemoglobin, Type A1C; FBG: fast blood glucose; HDL-C: high density lipoprotein cholesterol; TG: total cholesterol; LDL-C: low density lipoprotein cholesterol; Scr: serum creatinine; BUN: blood urea nitrogen; UA: uric acid; eGFR: estimated glomerular filtration rate; CKM: cardiovascular–kidney-metabolic; CTI: C-reactive protein-triglyceride-glucose index; CVD:cardiovascular disease; WC: waist circumference; WHtR: Waist-to-Height Ratio; WWI: Weight-adjusted waist index; BRI: Body Roundness Index; CVAI: Chinese Visceral Adiposity Index; CI: C-index.

**Table S10.** Associations between CTI and its related indices and CVD risk in CKM syndrome stage 0-2 stage

|  | Crude model | | Model 1 | | Model 2 | |
| --- | --- | --- | --- | --- | --- | --- |
|  | 95%CI | P | 95%CI | P | 95%CI | P |
| Continues CTI(per SD) | 1.17(1.09,1.25) | <0.0001 | 1.16(1.08,1.25) | <0.0001 | 1.13(1.05,1.22) | 0.002 |
| CTI |  |  |  |  |  |  |
| Q1 | ref |  | ref |  | ref |  |
| Q2 | 1.28(1.07,1.52) | 0.01 | 1.23(1.03,1.47) | 0.02 | 1.2(1.00,1.43) | 0.05 |
| Q3 | 1.42(1.19,1.70) | <0.001 | 1.35(1.13,1.62) | 0.001 | 1.3(1.08,1.57) | 0.01 |
| Q4 | 1.51(1.25,1.83) | <0.0001 | 1.48(1.22,1.79) | <0.0001 | 1.38(1.12,1.71) | 0.003 |
| P for trend |  | <0.0001 |  | <0.0001 |  | 0.001 |
| Continues CTI- BMI  (per SD) | 1.26(1.18,1.33) | <0.0001 | 1.27(1.19,1.35) | <0.0001 | 1.28(1.19,1.37) | <0.0001 |
| CTI-BMI |  |  |  |  |  |  |
| Q1 | ref |  | ref |  | ref |  |
| Q2 | 1.25(1.04,1.51) | 0.02 | 1.29(1.07,1.55) | 0.01 | 1.28(1.06,1.55) | 0.01 |
| Q3 | 1.39(1.15,1.68) | <0.001 | 1.44(1.19,1.74) | <0.001 | 1.43(1.17,1.75) | <0.001 |
| Q4 | 1.8(1.50,2.17) | <0.0001 | 1.89(1.56,2.29) | <0.0001 | 1.88(1.52,2.33) | <0.0001 |
| P for trend |  | <0.0001 |  | <0.0001 |  | <0.0001 |
| Continues CTI-WC  (per SD) | 1.27(1.19,1.37) | <0.0001 | 1.27(1.18,1.36) | <0.0001 | 1.27(1.17,1.38) | <0.0001 |
| CTI-WC |  |  |  |  |  |  |
| Q1 | ref |  | ref |  | ref |  |
| Q2 | 1.4(1.16,1.68) | <0.001 | 1.37(1.14,1.65) | <0.001 | 1.37(1.14,1.66) | <0.001 |
| Q3 | 1.63(1.36,1.97) | <0.0001 | 1.59(1.32,1.91) | <0.0001 | 1.59(1.31,1.93) | <0.0001 |
| Q4 | 1.89(1.56,2.29) | <0.0001 | 1.87(1.54,2.27) | <0.0001 | 1.88(1.52,2.33) | <0.0001 |
| P for trend |  | <0.0001 |  | <0.0001 |  | <0.0001 |
| Continues CTI-WHtR  (per SD) | 1(1.00,1.00) | <0.0001 | 1(1.00,1.00) | <0.0001 | 1(1.00,1.00) | <0.0001 |
| CTI-WHtR |  |  |  |  |  |  |
| Q1 | ref |  | ref |  | ref |  |
| Q2 | 1.32(1.09,1.59) | 0.004 | 1.28(1.06,1.54) | 0.01 | 1.27(1.05,1.54) | 0.01 |
| Q3 | 1.63(1.36,1.96) | <0.0001 | 1.55(1.29,1.87) | <0.0001 | 1.54(1.27,1.87) | <0.0001 |
| Q4 | 1.86(1.54,2.25) | <0.0001 | 1.76(1.45,2.14) | <0.0001 | 1.73(1.39,2.15) | <0.0001 |
| P for trend |  | <0.0001 |  | <0.0001 |  | <0.0001 |
| Continues CTI-WWI  (per SD) | 1.23(1.14,1.32) | <0.0001 | 1.17(1.09,1.27) | <0.0001 | 1.14(1.05,1.24) | 0.001 |
| CTI-WWI |  |  |  |  |  |  |
| Q1 | ref |  | ref |  | ref |  |
| Q2 | 1.36(1.14,1.63) | <0.001 | 1.29(1.08,1.55) | 0.01 | 1.27(1.06,1.52) | 0.01 |
| Q3 | 1.55(1.29,1.85) | <0.0001 | 1.43(1.19,1.71) | <0.001 | 1.37(1.13,1.65) | 0.001 |
| Q4 | 1.64(1.35,1.99) | <0.0001 | 1.47(1.21,1.80) | <0.001 | 1.38(1.11,1.72) | 0.004 |
| P for trend |  | <0.0001 |  | <0.0001 |  | 0.002 |
| Continues CTI-BRI  (per SD) | 1.28(1.20,1.37) | <0.0001 | 1.26(1.17,1.35) | <0.0001 | 1.25(1.16,1.35) | <0.0001 |
| CTI-BRI |  |  |  |  |  |  |
| Q1 | ref |  | ref |  | ref |  |
| Q2 | 1.29(1.07,1.57) | 0.01 | 1.28(1.06,1.55) | 0.01 | 1.27(1.05,1.55) | 0.01 |
| Q3 | 1.54(1.28,1.86) | <0.0001 | 1.49(1.23,1.80) | <0.0001 | 1.47(1.20,1.79) | <0.001 |
| Q4 | 1.92(1.59,2.31) | <0.0001 | 1.82(1.49,2.22) | <0.0001 | 1.78(1.43,2.20) | <0.0001 |
| P for trend |  | <0.0001 |  | <0.0001 |  | <0.0001 |
| Continues CTI-CVAI  (per SD) | 1.33(1.24,1.44) | <0.0001 | 1.29(1.19,1.39) | <0.0001 | 1.33(1.21,1.46) | <0.0001 |
| CTI-CVAI |  |  |  |  |  |  |
| Q1 | ref |  | ref |  | ref |  |
| Q2 | 1.43(1.20,1.72) | <0.001 | 1.33(1.10,1.60) | 0.003 | 1.34(1.11,1.63) | 0.002 |
| Q3 | 1.76(1.46,2.11) | <0.0001 | 1.6(1.33,1.93) | <0.0001 | 1.64(1.34,2.01) | <0.0001 |
| Q4 | 1.98(1.63,2.41) | <0.0001 | 1.81(1.48,2.22) | <0.0001 | 1.89(1.50,2.40) | <0.0001 |
| P for trend |  | <0.0001 |  | <0.0001 |  | <0.0001 |
| Continues CTI-CI  (per SD) | 1.23(1.14,1.32) | <0.0001 | 1.19(1.10,1.28) | <0.0001 | 1.16(1.07,1.26) | <0.001 |
| CTI-CI |  |  |  |  |  |  |
| Q1 | ref |  | ref |  | ref |  |
| Q2 | 1.31(1.10,1.57) | 0.003 | 1.26(1.06,1.51) | 0.01 | 1.24(1.04,1.49) | 0.02 |
| Q3 | 1.49(1.25,1.78) | <0.0001 | 1.4(1.17,1.67) | <0.001 | 1.35(1.12,1.63) | 0.002 |
| Q4 | 1.66(1.37,2.01) | <0.0001 | 1.52(1.25,1.85) | <0.0001 | 1.44(1.17,1.79) | <0.001 |
| P for trend |  | <0.0001 |  | <0.0001 |  | <0.001 |
| Columative CTI  (per SD) | 1.25(1.12,1.39) | <0.0001 | 1.25(1.12,1.40) | <0.0001 | 1.19(1.05,1.35) | 0.01 |
| Cluster CTI |  |  |  |  |  |  |
| low | ref |  | ref |  | ref |  |
| medium | 1.39(1.10,1.76) | 0.01 | 1.37(1.08,1.73) | 0.01 | 1.29(1.01,1.64) | 0.04 |
| high | 1.64(1.22,2.22) | 0.001 | 1.68(1.24,2.27) | <0.001 | 1.45(1.04,2.02) | 0.03 |
| P for trend |  | <0.001 |  | <0.001 |  | 0.02 |
| Columative CTI-BMI  (per SD) | 1.32(1.20,1.46) | <0.0001 | 1.36(1.23,1.51) | <0.0001 | 1.33(1.19,1.49) | <0.0001 |
| Cluster BMI |  |  |  |  |  |  |
| low | ref |  | ref |  | ref |  |
| medium | 1.5(1.18,1.91) | <0.001 | 1.57(1.23,2.01) | <0.001 | 1.5(1.16,1.93) | 0.002 |
| high | 2.11(1.59,2.79) | <0.0001 | 2.3(1.72,3.08) | <0.0001 | 2.14(1.56,2.95) | <0.0001 |
| P for trend |  | <0.0001 |  | <0.0001 |  | <0.0001 |
| Columative CTI-WC  (per SD) | 1.34(1.20,1.49) | <0.0001 | 1.36(1.22,1.52) | <0.0001 | 1.33(1.17,1.50) | <0.0001 |
| Cluster CTI-WC |  |  |  |  |  |  |
| low | ref |  | ref |  | ref |  |
| medium | 1.38(1.08,1.75) | 0.01 | 1.39(1.09,1.77) | 0.01 | 1.33(1.03,1.71) | 0.03 |
| high | 2.08(1.56,2.76) | <0.0001 | 2.14(1.60,2.85) | <0.0001 | 1.96(1.42,2.70) | <0.0001 |
| P for trend |  | <0.0001 |  | <0.0001 |  | <0.0001 |
| Columative CTI-WHtR  (per SD) | 1.36(1.22,1.52) | <0.0001 | 1.36(1.21,1.52) | <0.0001 | 1.32(1.16,1.50) | 1.36(1.22,1.52) |
| Cluster CTI-WHtR |  |  |  |  |  |  |
| low | ref |  | ref |  | ref |  |
| medium | 1.64(1.28,2.11) | <0.0001 | 1.62(1.26,2.09) | <0.001 | 1.53(1.18,1.99) | 0.001 |
| high | 2.1(1.57,2.81) | <0.0001 | 2.06(1.53,2.79) | <0.0001 | 1.85(1.32,2.58) | <0.001 |
| P for trend |  | <0.0001 |  | <0.0001 |  | <0.001 |
| Columative CTI-WWI  (per SD) | 1.32(1.18,1.48) | <0.0001 | 1.28(1.13,1.44) | <0.0001 | 1.22(1.06,1.39) | 0.004 |
| Cluster CTI-WWI |  |  |  |  |  |  |
| low | ref |  | ref |  | ref |  |
| medium | 1.32(1.01,1.72) | 0.04 | 1.27(0.97,1.66) | 0.08 | 1.19(0.90,1.57) | 0.22 |
| high | 1.97(1.47,2.65) | <0.0001 | 1.82(1.34,2.47) | <0.001 | 1.59(1.14,2.23) | 0.01 |
| P for trend |  | <0.0001 |  | <0.001 |  | 0.01 |
| Columative CTI-BRI  (per SD) | 1.36(1.23,1.51) | <0.0001 | 1.36(1.22,1.51) | <0.0001 | 1.32(1.17,1.48) | <0.0001 |
| Cluster CTI-BRI |  |  |  |  |  |  |
| low | ref |  | ref |  | ref |  |
| medium | 1.51(1.18,1.92) | <0.001 | 1.48(1.15,1.90) | 0.002 | 1.39(1.07,1.81) | 0.01 |
| high | 2.16(1.63,2.87) | <0.0001 | 2.12(1.57,2.86) | <0.0001 | 1.91(1.38,2.64) | <0.0001 |
| P for trend |  | <0.0001 |  | <0.0001 |  | <0.0001 |
| Columative CTI-CVAI  (per SD) | 1.4(1.25,1.57) | <0.0001 | 1.39(1.24,1.56) | <0.0001 | 1.37(1.20,1.57) | <0.0001 |
| Cluster CTI-CVAI |  |  |  |  |  |  |
| low | ref |  | ref |  | ref |  |
| medium | 1.82(1.42,2.34) | <0.0001 | 1.72(1.33,2.23) | <0.0001 | 1.66(1.26,2.19) | <0.001 |
| high | 2.34(1.72,3.17) | <0.0001 | 2.29(1.68,3.12) | <0.0001 | 2.19(1.54,3.11) | <0.0001 |
| P for trend |  | <0.0001 |  | <0.0001 |  | <0.0001 |
| Columative CTI-CI  (per SD) | 1.31(1.17,1.47) | <0.0001 | 1.29(1.14,1.45) | <0.0001 | 1.23(1.08,1.40) | 0.002 |
| Cluster CTI-CI |  |  |  |  |  |  |
| low | ref |  | ref |  | ref |  |
| medium | 1.46(1.12,1.91) | 0.01 | 1.43(1.09,1.87) | 0.01 | 1.33(1.01,1.76) | 0.04 |
| high | 1.91(1.41,2.58) | <0.0001 | 1.8(1.32,2.45) | <0.001 | 1.58(1.13,2.20) | 0.01 |
| P for trend |  | <0.0001 |  | <0.001 |  | 0.01 |

HR Hazard Ratio, CI Confidence Interval

Crude model: unadjusted for covariates;

Model 1: age, sex, marital status, education, eGFR, smoke status, drink status, residence place;

Model 2: age, sex, marital status, education, eGFR, smoke status, drink status, residence place, BUN, UA, LDL-C, HDL-C, HbA1c.

CTI: C-reactive protein-triglyceride-glucose index; CKM: cardiovascular–kidney–metabolic; CVD: cardiovascular disease; WC: waist circumference; WHtR: Waist-to-Height Ratio; WWI: Weight-adjusted waist index; BRI: Body Roundness Index; CVAI: Chinese Visceral Adiposity Index; CI: C-index

**Table S11.** Associations between CTI and its related indices and CVD risk in CKM syndrome stage 3

|  | Crude model | | Model 1 | | Model 2 | |
| --- | --- | --- | --- | --- | --- | --- |
|  | 95%CI | P | 95%CI | P | 95%CI | P |
| Continues CTI(per SD) | 1.12(1.04,1.20) | 0.002 | 1.09(1.02,1.18) | 0.02 | 1.09(1.00,1.19) | 0.04 |
| CTI |  |  |  |  |  |  |
| Q1 | ref |  | ref |  | ref |  |
| Q2 | 1.28(0.96,1.72) | 0.09 | 1.22(0.91,1.64) | 0.18 | 1.23(0.91,1.65) | 0.17 |
| Q3 | 1.6(1.22,2.09) | <0.001 | 1.51(1.14,1.98) | 0.003 | 1.52(1.15,2.02) | 0.004 |
| Q4 | 1.73(1.34,2.23) | <0.0001 | 1.59(1.22,2.08) | <0.001 | 1.63(1.22,2.17) | <0.001 |
| P for trend |  | <0.0001 |  | <0.001 |  | <0.001 |
| Continues CTI- BMI  (per SD) | 1.17(1.10,1.25) | <0.0001 | 1.16(1.08,1.24) | <0.0001 | 1.17(1.08,1.26) | <0.0001 |
| CTI-BMI |  |  |  |  |  |  |
| Q1 | ref |  | ref |  | ref |  |
| Q2 | 1.16(0.89,1.51) | 0.26 | 1.16(0.89,1.51) | 0.26 | 1.18(0.91,1.54) | 0.21 |
| Q3 | 1.3(1.02,1.66) | 0.03 | 1.31(1.02,1.69) | 0.03 | 1.36(1.04,1.76) | 0.02 |
| Q4 | 1.72(1.37,2.15) | <0.0001 | 1.67(1.31,2.13) | <0.0001 | 1.77(1.35,2.31) | <0.0001 |
| P for trend |  | <0.0001 |  | <0.0001 |  | <0.0001 |
| Continues CTI-WC  (per SD) | 1.17(1.09,1.26) | <0.0001 | 1.14(1.06,1.23) | <0.001 | 1.16(1.06,1.26) | <0.001 |
| CTI-WC |  |  |  |  |  |  |
| Q1 | ref |  | ref |  | ref |  |
| Q2 | 0.96(0.73,1.26) | 0.76 | 0.94(0.72,1.25) | 0.68 | 0.94(0.71,1.24) | 0.67 |
| Q3 | 1.28(1.00,1.63) | 0.05 | 1.22(0.95,1.57) | 0.12 | 1.24(0.95,1.60) | 0.11 |
| Q4 | 1.48(1.17,1.86) | <0.001 | 1.39(1.09,1.77) | 0.01 | 1.41(1.08,1.83) | 0.01 |
| P for trend |  | <0.0001 |  | <0.001 |  | 0.001 |
| Continues CTI-WHtR  (per SD) | 1(1.00,1.00) | <0.0001 | 1(1.00,1.00) | <0.001 | 1(1.00,1.00) | <0.001 |
| CTI-WHtR |  |  |  |  |  |  |
| Q1 | ref |  | ref |  | ref |  |
| Q2 | 1.09(0.83,1.43) | 0.53 | 1.1(0.84,1.45) | 0.49 | 1.11(0.85,1.47) | 0.44 |
| Q3 | 1.41(1.10,1.80) | 0.01 | 1.35(1.05,1.74) | 0.02 | 1.36(1.05,1.77) | 0.02 |
| Q4 | 1.65(1.31,2.07) | <0.0001 | 1.54(1.20,1.97) | <0.001 | 1.58(1.21,2.06) | <0.001 |
| P for trend |  | <0.0001 |  | <0.001 |  | <0.001 |
| Continues CTI-WWI  (per SD) | 1.11(1.04,1.20) | 0.004 | 1.06(0.98,1.15) | 0.13 | 1.05(0.96,1.14) | 0.27 |
| CTI-WWI |  |  |  |  |  |  |
| Q1 | ref |  | ref |  | ref |  |
| Q2 | 1.08(0.82,1.43) | 0.59 | 1.05(0.79,1.40) | 0.72 | 1.04(0.78,1.38) | 0.78 |
| Q3 | 1.35(1.04,1.75) | 0.02 | 1.26(0.97,1.64) | 0.08 | 1.25(0.96,1.64) | 0.10 |
| Q4 | 1.57(1.23,2.00) | <0.001 | 1.4(1.09,1.81) | 0.01 | 1.4(1.06,1.84) | 0.02 |
| P for trend |  | <0.0001 |  | 0.002 |  | 0.005 |
| Continues CTI-BRI  (per SD) | <0.0001 | 1.12(1.04,1.21) | 0.002 | 1.12(1.04,1.21) | 0.004 | <0.0001 |
| CTI-BRI |  |  |  |  |  |  |
| Q1 | ref |  | ref |  | ref |  |
| Q2 | 1(0.77,1.29) | 1.00 | 1(0.77,1.29) | 0.98 | 0.99(0.76,1.29) | 0.95 |
| Q3 | 1.32(1.04,1.68) | 0.02 | 1.25(0.98,1.60) | 0.07 | 1.26(0.98,1.62) | 0.07 |
| Q4 | 1.6(1.28,1.99) | <0.0001 | 1.47(1.16,1.87) | 0.001 | 1.5(1.16,1.94) | 0.002 |
| P for trend |  | <0.0001 |  | <0.001 |  | <0.001 |
| Continues CTI-CVAI  (per SD) | 1.2(1.12,1.28) | <0.0001 | 1.15(1.07,1.24) | <0.001 | 1.19(1.09,1.30) | <0.001 |
| CTI-CVAI |  |  |  |  |  |  |
| Q1 | ref |  | ref |  | ref |  |
| Q2 | 1.05(0.78,1.41) | 0.73 | 1.03(0.76,1.38) | 0.86 | 1.03(0.76,1.39) | 0.85 |
| Q3 | 1.31(1.01,1.71) | 0.04 | 1.22(0.93,1.60) | 0.15 | 1.26(0.94,1.68) | 0.12 |
| Q4 | 1.67(1.31,2.13) | <0.0001 | 1.5(1.16,1.93) | 0.002 | 1.6(1.20,2.15) | 0.002 |
| P for trend |  | <0.0001 |  | <0.001 |  | <0.001 |
| Continues CTI-CI  (per SD) | 1.12(1.04,1.20) | 0.003 | 1.08(1.00,1.16) | 0.06 | 1.07(0.98,1.16) | 0.13 |
| CTI-CI |  |  |  |  |  |  |
| Q1 | ref |  | ref |  | ref |  |
| Q2 | 1(0.75,1.33) | 0.98 | 0.95(0.71,1.28) | 0.76 | 0.95(0.71,1.27) | 0.72 |
| Q3 | 1.45(1.12,1.87) | 0.01 | 1.36(1.05,1.77) | 0.02 | 1.35(1.03,1.77) | 0.03 |
| Q4 | 1.55(1.21,1.98) | <0.001 | 1.39(1.07,1.79) | 0.01 | 1.39(1.06,1.83) | 0.02 |
| P for trend |  | <0.0001 |  | <0.001 |  | 0.002 |
| Columative CTI  (per SD) | 1.15(1.03,1.28) | 0.01 | - | - | - | - |
| Cluster CTI |  |  |  |  |  |  |
| low | ref |  | ref |  | ref |  |
| medium | 1.45(1.04,2.03) | 0.03 | 1.31(0.92,1.84) | 0.13 | 1.28(0.90,1.83) | 0.18 |
| high | 1.58(1.12,2.24) | 0.01 | 1.41(0.97,2.05) | 0.07 | 1.38(0.92,2.08) | 0.12 |
| P for trend |  | 0.01 |  | 0.08 |  | 0.14 |
| Columative CTI-BMI  (per SD) | 1.22(1.09,1.36) | <0.001 | - | - | - | - |
| Cluster BMI |  |  |  |  |  |  |
| low | ref |  | ref |  | ref |  |
| medium | 1.26(0.94,1.69) | 0.12 | 1.18(0.87,1.60) | 0.30 | 1.16(0.84,1.61) | 0.36 |
| high | 1.53(1.11,2.10) | 0.01 | 1.38(0.96,1.97) | 0.08 | 1.34(0.91,1.98) | 0.14 |
| P for trend |  | 0.01 |  | 0.08 |  | 0.14 |
| Columative CTI-WC  (per SD) | 1.19(1.06,1.33) | 0.003 | 1.15(1.01,1.30) | 0.03 | 1.15(1.00,1.32) | 0.05 |
| Cluster CTI-WC |  |  |  |  |  |  |
| low | ref |  | ref |  | ref |  |
| medium | 1.18(0.85,1.64) | 0.32 | 1.12(0.80,1.57) | 0.52 | 1.12(0.79,1.60) | 0.52 |
| high | 1.82(1.31,2.52) | <0.001 | 1.68(1.17,2.40) | 0.005 | 1.7(1.15,2.51) | 0.01 |
| P for trend |  | <0.001 |  | 0.002 |  | 0.004 |
| Columative CTI-WHtR  (per SD) | 1.19(1.07,1.33) | 0.002 | 1.13(1.00,1.28) | 0.06 | - | - |
| Cluster CTI-WHtR |  |  |  |  |  |  |
| low | ref |  | ref |  | ref |  |
| medium | 1.21(0.88,1.65) | 0.24 | 1.13(0.82,1.57) | 0.46 | 1.13(0.80,1.59) | 0.48 |
| high | 1.65(1.20,2.27) | 0.002 | 1.45(1.01,2.06) | 0.04 | 1.45(0.98,2.13) | 0.06 |
| P for trend |  | 0.002 |  | 0.04 |  | 0.05 |
| Columative CTI-WWI  (per SD) | 1.15(1.03,1.28) | 0.02 | - | - | - | - |
| Cluster CTI-WWI |  |  |  |  |  |  |
| low | ref |  | ref |  | ref |  |
| medium | 1.36(0.92,2.01) | 0.13 | 1.29(0.87,1.92) | 0.21 | 1.27(0.84,1.90) | 0.26 |
| high | 1.65(1.12,2.43) | 0.01 | 1.44(0.95,2.18) | 0.09 | 1.41(0.91,2.18) | 0.13 |
| P for trend |  | 0.01 |  | 0.1 |  | 0.14 |
| Columative CTI-BRI  (per SD) | 1.21(1.09,1.34) | <0.001 | 1.14(1.01,1.29) | 0.03 | 1.13(0.99,1.29) | 0.06 |
| Cluster CTI-BRI |  |  |  |  |  |  |
| low | ref |  | ref |  | ref |  |
| medium | 1.18(0.88,1.59) | 0.27 | 1.1(0.80,1.50) | 0.56 | 1.1(0.79,1.53) | 0.58 |
| high | 1.9(1.39,2.59) | <0.0001 | 1.68(1.18,2.40) | 0.004 | 1.68(1.15,2.47) | 0.01 |
| P for trend |  | <0.0001 |  | 0.004 |  | 0.01 |
| Columative CTI-CVAI  (per SD) | 1.19(1.06,1.32) | 0.002 | 1.13(1.00,1.28) | 0.04 | 1.15(1.00,1.32) | 0.05 |
| Cluster CTI-CVAI |  |  |  |  |  |  |
| low | ref |  | ref |  | ref |  |
| medium | 1.44(1.02,2.05) | 0.04 | 1.33(0.92,1.92) | 0.13 | 1.37(0.93,2.01) | 0.11 |
| high | 1.84(1.31,2.59) | <0.001 | 1.65(1.14,2.40) | 0.01 | 1.73(1.14,2.63) | 0.01 |
| P for trend |  | <0.001 |  | 0.01 |  | 0.01 |
| Columative CTI-CI  (per SD) | 1.14(1.02,1.28) | 0.02 | - | - | - | - |
| Cluster CTI-CI |  |  |  |  |  |  |
| low | ref |  | ref |  | ref |  |
| medium | 1.11(0.75,1.64) | 0.62 | 1.06(0.71,1.57) | 0.78 | 1.04(0.69,1.56) | 0.85 |
| high | 1.46(1.00,2.14) | 0.05 | 1.28(0.85,1.92) | 0.23 | 1.24(0.81,1.91) | 0.32 |
| P for trend |  | 0.02 |  | 0.14 |  | 0.21 |

HR Hazard Ratio, CI Confidence Interval

Crude model: unadjusted for covariates;

Model 1: age, sex, marital status, education, eGFR, smoke status, drink status, residence place;

Model 2: age, sex, marital status, education, eGFR, smoke status, drink status, residence place, BUN, UA, LDL-C, HDL-C, HbA1c.

CTI: C-reactive protein-triglyceride-glucose index; CKM: cardiovascular–kidney–metabolic; CVD: cardiovascular disease; WC: waist circumference; WHtR: Waist-to-Height Ratio; WWI: Weight-adjusted waist index; BRI: Body Roundness Index; CVAI: Chinese Visceral Adiposity Index; CI: C-index

**Table S12.** Threshold effect analysis of CTI on CVD risk using a two-piecewise linear regression model

| variable | Crude Model | Model 1 | Model 2 |
| --- | --- | --- | --- |
| standard logistic regression | 1.376(1.268,1.493) <0.0001 | 1.321(1.215,1.436) <0.0001 | 1.235(1.120,1.362) <0.0001 |
| Log-likelihood value | -13943.624 | -13885.474 | -13870.479 |
| two-piecewise linear regression | IP = 5.171 | IP = 5.171 | IP = 5.171 |
| Log-likelihood value | -13935.377 | -13879.513 | -13864.479 |
| CTI < IP | 1.721(1.478,2.003) <0.0001 | 1.604(1.375,1.871) <0.0001 | 1.55(1.311,1.833) <0.0001 |
| CTI ≥ IP | 0.938(0.701,1.256) 0.667 | 0.958(0.715,1.282) 0.772 | 0.877(0.647,1.188) 0.396 |
| p for Log-likelihood ratio | <0.001 | <0.01 | <0.01 |

HR Hazard Ratio, CI Confidence Interval

Crude model: unadjusted for covariates;

Model 1: age, sex, marital status, education, eGFR, smoke status, drink status, residence place;

Model 2: age, sex, marital status, education, eGFR, smoke status, drink status, residence place, BUN, UA, LDL-C, HDL-C, HbA1c

CTI: C-reactive protein-triglyceride-glucose index; CVD:cardiovascular disease; WC: waist circumference; WHtR: Waist-to-Height Ratio; WWI: Weight-adjusted waist index; BRI: Body Roundness Index; CVAI: Chinese Visceral Adiposity Index; CI: C-index.

**Table S13.** Threshold effect analysis of CTI-BMI on CVD risk using a two-piecewise linear regression model

| variable | Crude Model | Model 1 | Model 2 |
| --- | --- | --- | --- |
| standard logistic regression | 1.009(1.007,1.010) <0.0001 | 1.009(1.007,1.010) <0.0001 | 1.008(1.006,1.010) <0.0001 |
| Log-likelihood value | -13927.387 | -13861.881 | -13848.42 |
| two-piecewise linear regression | IP = 139.111 | IP = 139.111 | IP = 139.111 |
| Log-likelihood value | -13924.614 | -13857.609 | -13844.972 |
| CTI < IP | 1.011(1.008,1.014) <0.0001 | 1.012(1.008,1.015) <0.0001 | 1.011(1.008,1.015) <0.0001 |
| CTI ≥ IP | 1.004(0.999,1.009) 0.141 | 1.003(0.998,1.008) 0.264 | 1.003(0.998,1.009) 0.229 |
| p for Log-likelihood ratio | 0.062 | 0.014 | 0.032 |

HR Hazard Ratio, CI Confidence Interval

Crude model: unadjusted for covariates;

Model 1: age, sex, marital status, education, eGFR, smoke status, drink status, residence place;

Model 2: age, sex, marital status, education, eGFR, smoke status, drink status, residence place, BUN, UA, LDL-C, HDL-C, HbA1c

CTI: C-reactive protein-triglyceride-glucose index; CVD:cardiovascular disease; WC: waist circumference; WHtR: Waist-to-Height Ratio; WWI: Weight-adjusted waist index; BRI: Body Roundness Index; CVAI: Chinese Visceral Adiposity Index; CI: C-index.

**Table S14.** ROC curve analysis of CTI and related indices.

| Time | Ref | New | Group | AUC_ref | AUC_new | AUC_diff | p |
| --- | --- | --- | --- | --- | --- | --- | --- |
| 2 | CTI | CTI-BMI | Overall | 0.5603 | 0.5418 | -0.0185 | 0.096 |
| 2 | CTI | CTI-BMI | CKM0 stage | 0.5866 | 0.5182 | -0.0685 | 0.200 |
| 2 | CTI | CTI-BMI | CKM3 stage | 0.5554 | 0.5358 | -0.0196 | 0.224 |
| 2 | CTI | CTI-BRI | Overall | 0.5603 | 0.5516 | -0.0087 | 0.544 |
| 2 | CTI | CTI-BRI | CKM3 stage | 0.5554 | 0.5600 | 0.00462 | 0.756 |
| 2 | CTI | CTI-BRI | CKM0 stage | 0.5866 | 0.5612 | -0.0255 | 0.648 |
| 2 | CTI | CTI-CI | CKM3 stage | 0.5554 | 0.5527 | -0.0027 | 0.888 |
| 2 | CTI | CTI-CI | Overall | 0.5603 | 0.5662 | 0.0058 | 0.544 |
| 2 | CTI | CTI-CI | CKM0 stage | 0.5866 | 0.5998 | 0.0131 | 0.836 |
| 2 | CTI | CTI-CVAI | Overall | 0.5603 | 0.5635 | 0.0032 | 0.908 |
| 2 | CTI | CTI-CVAI | CKM3 stage | 0.5554 | 0.5534 | -0.0020 | 0.936 |
| 2 | CTI | CTI-CVAI | CKM0 stage | 0.5866 | 0.5430 | -0.0437 | 0.488 |
| 2 | CTI | CTI-WC | Overall | 0.5603 | 0.5560 | -0.0044 | 0.512 |
| 2 | CTI | CTI-WC | CKM3 stage | 0.5554 | 0.5509 | -0.0045 | 0.668 |
| 2 | CTI | CTI-WC | CKM0 stage | 0.5866 | 0.5852 | -0.0015 | 0.848 |
| 2 | CTI | CTI-WHtR | Overall | 0.5603 | 0.5614 | 0.0010 | 0.968 |
| 2 | CTI | CTI-WHtR | CKM0 stage | 0.5866 | 0.5953 | 0.0087 | 0.976 |
| 2 | CTI | CTI-WHtR | CKM3 stage | 0.5554 | 0.5613 | 0.0059 | 0.796 |
| 2 | CTI | CTI-WWI | CKM3 stage | 0.5554 | 0.5628 | 0.0074 | 0.632 |
| 2 | CTI | CTI-WWI | Overall | 0.5603 | 0.5709 | 0.0106 | 0.320 |
| 2 | CTI | CTI-WWI | CKM0 stage | 0.5866 | 0.5982 | 0.0116 | 0.832 |
| 4 | CTI | CTI-BMI | CKM0 stage | 0.5376 | 0.4749 | -0.0628 | 0.168 |
| 4 | CTI | CTI-BMI | Overall | 0.5537 | 0.5493 | -0.0044 | 0.516 |
| 4 | CTI | CTI-BMI | CKM3 stage | 0.5471 | 0.5414 | -0.0057 | 0.536 |
| 4 | CTI | CTI-BRI | Overall | 0.5538 | 0.5513 | -0.0025 | 0.792 |
| 4 | CTI | CTI-BRI | CKM3 stage | 0.5472 | 0.5487 | 0.0015 | 0.948 |
| 4 | CTI | CTI-BRI | CKM0 stage | 0.5376 | 0.5569 | 0.0193 | 0.828 |
| 4 | CTI | CTI-CI | CKM3 stage | 0.5472 | 0.5366 | -0.0106 | 0.404 |
| 4 | CTI | CTI-CI | Overall | 0.5538 | 0.5568 | 0.0030 | 0.676 |
| 4 | CTI | CTI-CI | CKM0 stage | 0.5376 | 0.5833 | 0.0457 | 0.248 |
| 4 | CTI | CTI-CVAI | Overall | 0.5538 | 0.5647 | 0.0109 | 0.316 |
| 4 | CTI | CTI-CVAI | CKM3 stage | 0.5472 | 0.5469 | -0.0003 | 0.960 |
| 4 | CTI | CTI-CVAI | CKM0 stage | 0.5376 | 0.5281 | -0.0096 | 0.876 |
| 4 | CTI | CTI-WC | Overall | 0.5538 | 0.5535 | -0.0002 | 0.800 |
| 4 | CTI | CTI-WC | CKM3 stage | 0.5472 | 0.5428 | -0.0043 | 0.632 |
| 4 | CTI | CTI-WC | CKM0 stage | 0.5376 | 0.5584 | 0.0207 | 0.740 |
| 4 | CTI | CTI-WHtR | Overall | 0.5538 | 0.5570 | 0.0033 | 0.812 |
| 4 | CTI | CTI-WHtR | CKM3 stage | 0.5472 | 0.5483 | 0.0011 | 0.908 |
| 4 | CTI | CTI-WHtR | CKM0 stage | 0.5376 | 0.5889 | 0.0513 | 0.272 |
| 4 | CTI | CTI-WWI | CKM3 stage | 0.5472 | 0.5413 | -0.0059 | 0.688 |
| 4 | CTI | CTI-WWI | Overall | 0.5538 | 0.5591 | 0.0053 | 0.504 |
| 4 | CTI | CTI-WWI | CKM0 stage | 0.5376 | 0.5743 | 0.0367 | 0.388 |
| 7 | CTI | CTI-BMI | Overall | 0.5657 | 0.5667 | 0.0011 | 0.512 |
| 7 | CTI | CTI-BMI | CKM3 stage | 0.5542 | 0.5568 | 0.0026 | 0.788 |
| 7 | CTI | CTI-BMI | CKM0 stage | 0.5051 | 0.4854 | -0.0198 | 0.500 |
| 7 | CTI | CTI-BRI | Overall | 0.5657 | 0.5759 | 0.0102 | 0.528 |
| 7 | CTI | CTI-BRI | CKM0 stage | 0.5051 | 0.5865 | 0.0813 | 0.196 |
| 7 | CTI | CTI-BRI | CKM3 stage | 0.5542 | 0.5692 | 0.0150 | 0.392 |
| 7 | CTI | CTI-CI | Overall | 0.5657 | 0.5755 | 0.0099 | 0.092 |
| 7 | CTI | CTI-CI | CKM3 stage | 0.5542 | 0.5561 | 0.0019 | 0.816 |
| 7 | CTI | CTI-CI | CKM0 stage | 0.5051 | 0.5473 | 0.0421 | 0.216 |
| 7 | CTI | CTI-CVAI | Overall | 0.5657 | 0.5830 | 0.0173 | 0.080 |
| 7 | CTI | CTI-CVAI | CKM3 stage | 0.5542 | 0.5674 | 0.0132 | 0.484 |
| 7 | CTI | CTI-CVAI | CKM0 stage | 0.5051 | 0.5744 | 0.0693 | 0.272 |
| 7 | CTI | CTI-WC | Overall | 0.5657 | 0.5757 | 0.0100 | 0.352 |
| 7 | CTI | CTI-WC | CKM3 stage | 0.5542 | 0.5677 | 0.0135 | 0.424 |
| 7 | CTI | CTI-WC | CKM0 stage | 0.5051 | 0.5338 | 0.0287 | 0.608 |
| 7 | CTI | CTI-WHtR | Overall | 0.5657 | 0.5781 | 0.0125 | 0.152 |
| 7 | CTI | CTI-WHtR | CKM3 stage | 0.5542 | 0.5716 | 0.0173 | 0.200 |
| 7 | CTI | CTI-WHtR | CKM0 stage | 0.5051 | 0.5604 | 0.0552 | 0.116 |
| 7 | CTI | CTI-WWI | Overall | 0.5657 | 0.5775 | 0.0118 | 0.068 |
| 7 | CTI | CTI-WWI | CKM0 stage | 0.5051 | 0.5438 | 0.0386 | 0.240 |
| 7 | CTI | CTI-WWI | CKM3 stage | 0.5542 | 0.5609 | 0.0067 | 0.572 |
| 9 | CTI | CTI-BMI | Overall | 0.5679 | 0.5711 | 0.0032 | 0.828 |
| 9 | CTI | CTI-BMI | CKM3 stage | 0.5517 | 0.5653 | 0.0137 | 0.392 |
| 9 | CTI | CTI-BMI | CKM0 stage | 0.5236 | 0.5073 | -0.0163 | 0.544 |
| 9 | CTI | CTI-BRI | Overall | 0.5679 | 0.5741 | 0.0062 | 0.800 |
| 9 | CTI | CTI-BRI | CKM3 stage | 0.5517 | 0.5665 | 0.0149 | 0.292 |
| 9 | CTI | CTI-BRI | CKM0 stage | 0.5236 | 0.5480 | 0.0244 | 0.648 |
| 9 | CTI | CTI-CI | Overall | 0.5679 | 0.5750 | 0.0071 | 0.148 |
| 9 | CTI | CTI-CI | CKM3 stage | 0.5517 | 0.5557 | 0.0040 | 0.732 |
| 9 | CTI | CTI-CI | CKM0 stage | 0.5236 | 0.5490 | 0.0254 | 0.440 |
| 9 | CTI | CTI-CVAI | Overall | 0.5679 | 0.5849 | 0.0170 | 0.076 |
| 9 | CTI | CTI-CVAI | CKM3 stage | 0.5517 | 0.5720 | 0.0203 | 0.140 |
| 9 | CTI | CTI-CVAI | CKM0 stage | 0.5236 | 0.5480 | 0.0244 | 0.608 |
| 9 | CTI | CTI-WC | Overall | 0.5679 | 0.5785 | 0.0106 | 0.204 |
| 9 | CTI | CTI-WC | CKM3 stage | 0.5517 | 0.5690 | 0.0173 | 0.256 |
| 9 | CTI | CTI-WC | CKM0 v | 0.5236 | 0.5378 | 0.0142 | 0.788 |
| 9 | CTI | CTI-WHtR | Overall | 0.5679 | 0.5790 | 0.0111 | 0.180 |
| 9 | CTI | CTI-WHtR | CKM3 stage | 0.5517 | 0.5702 | 0.0185 | 0.120 |
| 9 | CTI | CTI-WHtR | CKM0 stage | 0.5236 | 0.5527 | 0.0291 | 0.436 |
| 9 | CTI | CTI-WWI | Overall | 0.5679 | 0.5751 | 0.0072 | 0.264 |
| 9 | CTI | CTI-WWI | CKM0 stage | 0.5236 | 0.5447 | 0.0211 | 0.412 |
| 9 | CTI | CTI-WWI | CKM3 stage | 0.5517 | 0.5584 | 0.0067 | 0.564 |

AUC: area under curve; ROC: receiver operating characteristic

**Table S15.** NRI and IDI curve analysis of CTI and related indices.

| Time | Ref | New | Group | cNRI | cNRI_p | IDI | IDI_p |
| --- | --- | --- | --- | --- | --- | --- | --- |
| 2 | CTI | CTI-BMI | Overall | 0.0182 | 0.728 | -0.0096 | 0.380 |
| 2 | CTI | CTI-BMI | CKM0 stage | -0.0734 | 0.756 | -0.0559 | 0.304 |
| 2 | CTI | CTI-BMI | CKM3 stage | -0.0014 | 0.984 | -0.0035 | 0.804 |
| 2 | CTI | CTI-BRI | Overall | 0.0303 | 0.568 | 0.0020 | 0.912 |
| 2 | CTI | CTI-BRI | CKM3 stage | 0.0403 | 0.600 | 0.0218 | 0.296 |
| 2 | CTI | CTI-BRI | CKM0 stage | -0.0609 | 0.788 | -0.0419 | 0.484 |
| 2 | CTI | CTI-CI | CKM3 stage | 0.1359 | 0.060 | 0.0135 | 0.164 |
| 2 | CTI | CTI-CI | Overall | 0.0618 | 0.216 | 0.0064 | 0.376 |
| 2 | CTI | CTI-CI | CKM0 stage | 0.0344 | 0.952 | -0.0005 | 0.964 |
| 2 | CTI | CTI-CVAI | Overall | 0.0287 | 0.616 | 0.0108 | 0.380 |
| 2 | CTI | CTI-CVAI | CKM3 stage | 0.0318 | 0.672 | 0.0217 | 0.244 |
| 2 | CTI | CTI-CVAI | CKM0 stage | -0.0930 | 0.688 | -0.0614 | 0.288 |
| 2 | CTI | CTI-WC | Overall | 0.0510 | 0.356 | 0.0028 | 0.780 |
| 2 | CTI | CTI-WC | CKM3 stage | 0.0300 | 0.676 | 0.0136 | 0.312 |
| 2 | CTI | CTI-WC | CKM0 stage | 0.0672 | 0.764 | 0.0013 | 0.988 |
| 2 | CTI | CTI-WHtR | Overall | 0.0622 | 0.260 | 0.0073 | 0.408 |
| 2 | CTI | CTI-WHtR | CKM0 stage | -0.2033 | 0.388 | -0.0146 | 0.708 |
| 2 | CTI | CTI-WHtR | CKM3 stage | 0.0585 | 0.412 | 0.0186 | 0.192 |
| 2 | CTI | CTI-WWI | CKM3 stage | 0.0817 | 0.296 | 0.0142 | 0.196 |
| 2 | CTI | CTI-WWI | Overall | 0.0465 | 0.356 | 0.0077 | 0.316 |
| 2 | CTI | CTI-WWI | CKM0 stage | -0.1240 | 0.600 | -0.0074 | 0.768 |
| 4 | CTI | CTI-BMI | CKM0 stage | -0.1681 | 0.452 | -0.0625 | 0.176 |
| 4 | CTI | CTI-BMI | Overall | 0.0113 | 0.812 | -0.0003 | 0.988 |
| 4 | CTI | CTI-BMI | CKM3 stage | 0.0094 | 0.844 | 0.0032 | 0.740 |
| 4 | CTI | CTI-BRI | Overall | 0.0242 | 0.532 | 0.0034 | 0.700 |
| 4 | CTI | CTI-BRI | CKM3 stage | 0.0126 | 0.776 | 0.0084 | 0.532 |
| 4 | CTI | CTI-BRI | CKM0 stage | -0.0236 | 0.908 | -0.0007 | 0.964 |
| 4 | CTI | CTI-CI | CKM3 stage | 0.1117 | 0.072 | 0.0033 | 0.644 |
| 4 | CTI | CTI-CI | Overall | 0.0535 | 0.208 | 0.0055 | 0.352 |
| 4 | CTI | CTI-CI | CKM0 stage | 0.0976 | 0.644 | 0.0260 | 0.356 |
| 4 | CTI | CTI-CVAI | Overall | 0.0815 | 0.052 | 0.0198 | 0.032 |
| 4 | CTI | CTI-CVAI | CKM3 stage | 0.0490 | 0.376 | 0.0190 | 0.240 |
| 4 | CTI | CTI-CVAI | CKM0 stage | 0.0011 | 0.956 | -0.0245 | 0.716 |
| 4 | CTI | CTI-WC | Overall | 0.0572 | 0.160 | 0.0078 | 0.308 |
| 4 | CTI | CTI-WC | CKM3 stage | 0.0434 | 0.384 | 0.0099 | 0.352 |
| 4 | CTI | CTI-WC | CKM0 stage | 0.1056 | 0.608 | 0.0104 | 0.712 |
| 4 | CTI | CTI-WHtR | Overall | 0.0538 | 0.208 | 0.0084 | 0.268 |
| 4 | CTI | CTI-WHtR | CKM3 stage | 0.0357 | 0.464 | 0.0086 | 0.428 |
| 4 | CTI | CTI-WHtR | CKM0 stage | -0.0974 | 0.668 | 0.0082 | 0.808 |
| 4 | CTI | CTI-WWI | CKM3 stage | -0.0352 | 0.588 | 0.0002 | 0.916 |
| 4 | CTI | CTI-WWI | Overall | 0.0073 | 0.856 | 0.0044 | 0.468 |
| 4 | CTI | CTI-WWI | CKM0 stage | -0.0129 | 0.968 | 0.0214 | 0.516 |
| 7 | CTI | CTI-BMI | Overall | 0.0530 | 0.092 | 0.0056 | 0.348 |
| 7 | CTI | CTI-BMI | CKM3 stage | 0.0361 | 0.492 | 0.0108 | 0.312 |
| 7 | CTI | CTI-BMI | CKM0 stage | -0.0235 | 0.812 | -0.0113 | 0.772 |
| 7 | CTI | CTI-BRI | Overall | 0.0705 | 0.024 | 0.0154 | 0.052 |
| 7 | CTI | CTI-BRI | CKM0 stage | 0.2158 | 0.140 | 0.0693 | 0.168 |
| 7 | CTI | CTI-BRI | CKM3 stage | 0.0599 | 0.212 | 0.0202 | 0.128 |
| 7 | CTI | CTI-CI | Overall | 0.1110 | <0.001 | 0.0112 | 0.024 |
| 7 | CTI | CTI-CI | CKM3 stage | 0.1193 | 0.020 | 0.0097 | 0.188 |
| 7 | CTI | CTI-CI | CKM0 stage | 0.1816 | 0.240 | 0.0371 | 0.052 |
| 7 | CTI | CTI-CVAI | Overall | 0.1303 | <0.001 | 0.0265 | <0.001 |
| 7 | CTI | CTI-CVAI | CKM3 stage | 0.0869 | 0.076 | 0.0237 | 0.040 |
| 7 | CTI | CTI-CVAI | CKM0 stage | 0.1709 | 0.292 | 0.0588 | 0.212 |
| 7 | CTI | CTI-WC | Overall | 0.1192 | <0.001 | 0.0150 | 0.020 |
| 7 | CTI | CTI-WC | CKM3 stage | 0.1402 | <0.001 | 0.0164 | 0.080 |
| 7 | CTI | CTI-WC | CKM0 stage | 0.2500 | 0.092 | 0.0315 | 0.232 |
| 7 | CTI | CTI-WHtR | Overall | 0.1040 | 0.004 | 0.0162 | 0.016 |
| 7 | CTI | CTI-WHtR | CKM3 stage | 0.1097 | 0.020 | 0.0164 | 0.096 |
| 7 | CTI | CTI-WHtR | CKM0 stage | 0.1068 | 0.476 | 0.0468 | 0.104 |
| 7 | CTI | CTI-WWI | Overall | 0.0561 | 0.072 | 0.0102 | 0.048 |
| 7 | CTI | CTI-WWI | CKM0 stage | 0.1581 | 0.276 | 0.0438 | 0.044 |
| 7 | CTI | CTI-WWI | CKM3 stage | 0.0246 | 0.604 | 0.0070 | 0.376 |
| 9 | CTI | CTI-BMI | Overall | 0.0671 | 0.012 | 0.0084 | 0.160 |
| 9 | CTI | CTI-BMI | CKM3 stage | 0.0916 | 0.056 | 0.0200 | 0.036 |
| 9 | CTI | CTI-BMI | CKM0 stage | -0.0654 | 0.604 | -0.0155 | 0.612 |
| 9 | CTI | CTI-BRI | Overall | 0.0548 | 0.024 | 0.0137 | 0.104 |
| 9 | CTI | CTI-BRI | CKM3 stage | 0.0609 | 0.168 | 0.0228 | 0.096 |
| 9 | CTI | CTI-BRI | CKM0 stage | 0.0259 | 0.844 | 0.0083 | 0.832 |
| 9 | CTI | CTI-CI | Overall | 0.0970 | <0.001 | 0.0088 | 0.060 |
| 9 | CTI | CTI-CI | CKM3 stage | 0.0763 | 0.104 | 0.0068 | 0.384 |
| 9 | CTI | CTI-CI | CKM0 stage | 0.0002 | 0.972 | 0.0131 | 0.536 |
| 9 | CTI | CTI-CVAI | Overall | 0.1261 | <0.001 | 0.0264 | <0.001 |
| 9 | CTI | CTI-CVAI | CKM3 stage | 0.1085 | 0.012 | 0.0287 | 0.020 |
| 9 | CTI | CTI-CVAI | CKM0 stage | 0.1138 | 0.364 | 0.0129 | 0.756 |
| 9 | CTI | CTI-WC | Overall | 0.1137 | <0.001 | 0.0147 | 0.012 |
| 9 | CTI | CTI-WC | CKM3 stage | 0.1368 | 0.004 | 0.0182 | 0.040 |
| 9 | CTI | CTI-WC | CKM0 v | 0.0841 | 0.524 | 0.0076 | 0.768 |
| 9 | CTI | CTI-WHtR | Overall | 0.0893 | <0.001 | 0.0147 | 0.016 |
| 9 | CTI | CTI-WHtR | CKM3 stage | 0.1153 | 0.012 | 0.0178 | 0.080 |
| 9 | CTI | CTI-WHtR | CKM0 stage | -0.0478 | 0.684 | 0.0120 | 0.668 |
| 9 | CTI | CTI-WWI | Overall | 0.0484 | 0.080 | 0.0072 | 0.148 |
| 9 | CTI | CTI-WWI | CKM0 stage | 0.0297 | 0.808 | 0.0140 | 0.496 |
| 9 | CTI | CTI-WWI | CKM3 stage | 0.0056 | 0.916 | 0.0050 | 0.536 |

**Table S16.** ROC curve analysis of cumulative CTI and related indices.

| Time | Ref | New | Group | AUC_ref | AUC_new | AUC_diff | p |
| --- | --- | --- | --- | --- | --- | --- | --- |
| 3 | cumulative CTI | cumulative CTI-BMI | CKM0 stage | 0.4302 | 0.4144 | -0.0157 | 0.800 |
| 3 | cumulative CTI | cumulative CTI-BMI | CKM3 stage | 0.5559 | 0.5476 | -0.0083 | 0.720 |
| 3 | cumulative CTI | cumulative CTI-BMI | Overall | 0.5662 | 0.5644 | -0.0018 | 0.868 |
| 3 | cumulative CTI | cumulative CTI-BRI | CKM0 stage | 0.4302 | 0.4676 | 0.0375 | 0.864 |
| 3 | cumulative CTI | cumulative CTI-BRI | CKM3 stage | 0.5559 | 0.5748 | 0.0189 | 0.364 |
| 3 | cumulative CTI | cumulative CTI-BRI | Overall | 0.5662 | 0.5774 | 0.0112 | 0.496 |
| 3 | cumulative CTI | cumulative CTI-CI | CKM0 stage | 0.4302 | 0.3629 | -0.0673 | 0.244 |
| 3 | cumulative CTI | cumulative CTI-CI | CKM3 stage | 0.5559 | 0.5705 | 0.0146 | 0.284 |
| 3 | cumulative CTI | cumulative CTI-CI | Overall | 0.5662 | 0.5874 | 0.0212 | 0.032 |
| 3 | cumulative CTI | cumulative CTI-CVAI | CKM0 stage | 0.4302 | 0.5176 | 0.0875 | 0.612 |
| 3 | cumulative CTI | cumulative CTI-CVAI | CKM3 stage | 0.5559 | 0.5668 | 0.0110 | 0.604 |
| 3 | cumulative CTI | cumulative CTI-CVAI | Overall | 0.5662 | 0.5833 | 0.0172 | 0.160 |
| 3 | cumulative CTI | cumulative CTI-WC | CKM0 stage | 0.4302 | 0.3046 | -0.1256 | 0.264 |
| 3 | cumulative CTI | cumulative CTI-WC | CKM3 stage | 0.5559 | 0.5702 | 0.014 | 0.372 |
| 3 | cumulative CTI | cumulative CTI-WC | Overall | 0.5662 | 0.5788 | 0.0126 | 0.244 |
| 3 | cumulative CTI | cumulative CTI-WHtR | CKM0 stage | 0.4302 | 0.4592 | 0.0290 | 0.888 |
| 3 | cumulative CTI | cumulative CTI-WHtR | CKM3 stage | 0.5559 | 0.5727 | 0.0168 | 0.352 |
| 3 | cumulative CTI | cumulative CTI-WHtR | Overall | 0.5662 | 0.5784 | 0.0123 | 0.312 |
| 3 | cumulative CTI | cumulative CTI-WWI | CKM0 stage | 0.4302 | 0.4194 | -0.0108 | 0.952 |
| 3 | cumulative CTI | cumulative CTI-WWI | CKM3 stage | 0.5559 | 0.5646 | 0.0087 | 0.436 |
| 3 | cumulative CTI | cumulative CTI-WWI | Overall | 0.5662 | 0.5829 | 0.0167 | 0.092 |
| 5 | cumulative CTI | cumulative CTI-BMI | CKM0 stage | 0.4173 | 0.4062 | -0.0110 | 0.604 |
| 5 | cumulative CTI | cumulative CTI-BMI | CKM3 stage | 0.5490 | 0.5563 | 0.0074 | 0.572 |
| 5 | cumulative CTI | cumulative CTI-BMI | Overall | 0.5710 | 0.5745 | 0.0034 | 0.740 |
| 5 | cumulative CTI | cumulative CTI-BRI | CKM0 stage | 0.4173 | 0.4396 | 0.0223 | 0.968 |
| 5 | cumulative CTI | cumulative CTI-BRI | CKM3 stage | 0.5490 | 0.5733 | 0.0244 | 0.232 |
| 5 | cumulative CTI | cumulative CTI-BRI | Overall | 0.5712 | 0.5762 | 0.0050 | 0.708 |
| 5 | cumulative CTI | cumulative CTI-CI | CKM0 stage | 0.4173 | 0.4113 | -0.0059 | 0.796 |
| 5 | cumulative CTI | cumulative CTI-CI | CKM3 stage | 0.5490 | 0.5641 | 0.0151 | 0.212 |
| 5 | cumulative CTI | cumulative CTI-CI | Overall | 0.5712 | 0.5829 | 0.0118 | 0.140 |
| 5 | cumulative CTI | cumulative CTI-CVAI | CKM0 stage | 0.4173 | 0.4984 | 0.0812 | 0.496 |
| 5 | cumulative CTI | cumulative CTI-CVAI | CKM3 stage | 0.5490 | 0.5711 | 0.0222 | 0.248 |
| 5 | cumulative CTI | cumulative CTI-CVAI | Overall | 0.5712 | 0.5882 | 0.0170 | 0.168 |
| 5 | cumulative CTI | cumulative CTI-WC | CKM0 stage | 0.4173 | 0.3636 | -0.0537 | 0.348 |
| 5 | cumulative CTI | cumulative CTI-WC | CKM3 stage | 0.5490 | 0.5689 | 0.0200 | 0.160 |
| 5 | cumulative CTI | cumulative CTI-WC | Overall | 0.5712 | 0.5828 | 0.0116 | 0.256 |
| 5 | cumulative CTI | cumulative CTI-WHtR | CKM0 stage | 0.4173 | 0.4395 | 0.0223 | 0.892 |
| 5 | cumulative CTI | cumulative CTI-WHtR | CKM3 stage | 0.5490 | 0.5731 | 0.0241 | 0.132 |
| 5 | cumulative CTI | cumulative CTI-WHtR | Overall | 0.5712 | 0.5808 | 0.0097 | 0.340 |
| 5 | cumulative CTI | cumulative CTI-WWI | CKM0 stage | 0.4173 | 0.4374 | 0.0201 | 0.752 |
| 5 | cumulative CTI | cumulative CTI-WWI | CKM3 stage | 0.5490 | 0.5634 | 0.0145 | 0.280 |
| 5 | cumulative CTI | cumulative CTI-WWI | Overall | 0.5712 | 0.5788 | 0.0076 | 0.356 |

**Table S17.** NRI and IDI curve analysis of cumulative CTI and related indices.

| Time | Ref | New | Group | cNRI | cNRI_p | IDI | IDI_p |
| --- | --- | --- | --- | --- | --- | --- | --- |
| 3 | cumulative CTI | cumulative CTI-BMI | CKM0 stage | -0.084 | 0.775 | -0.0328 | 0.625 |
| 3 | cumulative CTI | cumulative CTI-BMI | CKM3 stage | -0.0113 | 0.892 | 0.0067 | 0.684 |
| 3 | cumulative CTI | cumulative CTI-BMI | Overall | 0.0120 | 0.820 | 0.0014 | 0.916 |
| 3 | cumulative CTI | cumulative CTI-BRI | CKM0 stage | -0.1280 | 0.686 | -0.0557 | 0.511 |
| 3 | cumulative CTI | cumulative CTI-BRI | CKM3 stage | -0.0553 | 0.492 | 0.0186 | 0.388 |
| 3 | cumulative CTI | cumulative CTI-BRI | Overall | -0.0021 | 0.948 | 0.0156 | 0.284 |
| 3 | cumulative CTI | cumulative CTI-CI | CKM0 stage | -0.2040 | 0.423 | -0.0567 | 0.239 |
| 3 | cumulative CTI | cumulative CTI-CI | CKM3 stage | 0.0478 | 0.552 | 0.0125 | 0.328 |
| 3 | cumulative CTI | cumulative CTI-CI | Overall | 0.1068 | 0.048 | 0.0146 | 0.088 |
| 3 | cumulative CTI | cumulative CTI-CVAI | CKM0 stage | -0.1200 | 0.8016 | -0.0058 | 0.978 |
| 3 | cumulative CTI | cumulative CTI-CVAI | CKM3 stage | 0.0712 | 0.404 | 0.0146 | 0.452 |
| 3 | cumulative CTI | cumulative CTI-CVAI | Overall | 0.1028 | 0.044 | 0.0240 | 0.064 |
| 3 | cumulative CTI | cumulative CTI-WC | CKM0 stage | -0.1553 | 0.550 | -0.0822 | 0.154 |
| 3 | cumulative CTI | cumulative CTI-WC | CKM3 stage | 0.0301 | 0.672 | 0.0146 | 0.368 |
| 3 | cumulative CTI | cumulative CTI-WC | Overall | 0.0555 | 0.276 | 0.0146 | 0.180 |
| 3 | cumulative CTI | cumulative CTI-WHtR | CKM0 stage | -0.1520 | 0.585 | -0.0528 | 0.394 |
| 3 | cumulative CTI | cumulative CTI-WHtR | CKM3 stage | -0.0280 | 0.768 | 0.0166 | 0.288 |
| 3 | cumulative CTI | cumulative CTI-WHtR | Overall | 0.0229 | 0.672 | 0.0161 | 0.168 |
| 3 | cumulative CTI | cumulative CTI-WWI | CKM0 stage | -0.1160 | 0.684 | -0.0342 | 0.499 |
| 3 | cumulative CTI | cumulative CTI-WWI | CKM3 stage | 0.0464 | 0.616 | 0.0100 | 0.476 |
| 3 | cumulative CTI | cumulative CTI-WWI | Overall | 0.0781 | 0.132 | 0.0138 | 0.124 |
| 5 | cumulative CTI | cumulative CTI-BMI | CKM0 stage | -0.3271 | 0.160 | -0.0490 | 0.256 |
| 5 | cumulative CTI | cumulative CTI-BMI | CKM3 stage | 0.0746 | 0.276 | 0.0205 | 0.152 |
| 5 | cumulative CTI | cumulative CTI-BMI | Overall | 0.0787 | 0.072 | 0.0078 | 0.320 |
| 5 | cumulative CTI | cumulative CTI-BRI | CKM0 stage | -0.2781 | 0.208 | -0.0847 | 0.180 |
| 5 | cumulative CTI | cumulative CTI-BRI | CKM3 stage | -0.0521 | 0.492 | 0.0188 | 0.316 |
| 5 | cumulative CTI | cumulative CTI-BRI | Overall | -0.0090 | 0.872 | 0.0099 | 0.332 |
| 5 | cumulative CTI | cumulative CTI-CI | CKM0 stage | -0.2152 | 0.320 | -0.0434 | 0.184 |
| 5 | cumulative CTI | cumulative CTI-CI | CKM3 stage | 0.0127 | 0.844 | 0.0086 | 0.488 |
| 5 | cumulative CTI | cumulative CTI-CI | Overall | 0.0707 | 0.128 | 0.0085 | 0.180 |
| 5 | cumulative CTI | cumulative CTI-CVAI | CKM0 stage | -0.0789 | 0.744 | -0.0314 | 0.684 |
| 5 | cumulative CTI | cumulative CTI-CVAI | CKM3 stage | 0.0835 | 0.196 | 0.0220 | 0.176 |
| 5 | cumulative CTI | cumulative CTI-CVAI | Overall | 0.0934 | 0.040 | 0.0237 | 0.016 |
| 5 | cumulative CTI | cumulative CTI-WC | CKM0 stage | -0.1713 | 0.436 | -0.0631 | 0.096 |
| 5 | cumulative CTI | cumulative CTI-WC | CKM3 stage | 0.0636 | 0.364 | 0.0175 | 0.236 |
| 5 | cumulative CTI | cumulative CTI-WC | Overall | 0.0247 | 0.560 | 0.0129 | 0.132 |
| 5 | cumulative CTI | cumulative CTI-WHtR | CKM0 stage | -0.2950 | 0.192 | -0.0578 | 0.156 |
| 5 | cumulative CTI | cumulative CTI-WHtR | CKM3 stage | -0.0060 | 0.992 | 0.0174 | 0.208 |
| 5 | cumulative CTI | cumulative CTI-WHtR | Overall | 0.0123 | 0.748 | 0.0120 | 0.132 |
| 5 | cumulative CTI | cumulative CTI-WWI | CKM0 stage | -0.2697 | 0.224 | -0.0360 | 0.260 |
| 5 | cumulative CTI | cumulative CTI-WWI | CKM3 stage | 0.0064 | 0.968 | 0.0068 | 0.584 |
| 5 | cumulative CTI | cumulative CTI-WWI | Overall | 0.0309 | 0.532 | 0.0063 | 0.372 |

**Table S18.** Baseline characteristics comparison between excluded and included participants

| **Variable** | **Excluded(N=4690, 39.7 %)** | **Included (N=7118, 60.3 %)** | **P-value** | **Effect_Size** |
| --- | --- | --- | --- | --- |
| Age | 59[52-67] | 58[51-64] | < 0.001 | -0.073 |
| Sbp | 130.33[116.67-146.67] | 126.67[114.33-141.33] | < 0.001 | -0.096 |
| Dbp | 75.67[68-84.67] | 74.67[67-83] | < 0.001 | -0.051 |
| TG | 113.28[79.65-168.15] | 102.66[73.46-148.68] | < 0.001 | -0.107 |
| FBG | 102.6[94.86-115.02] | 102.06[94.5-111.96] | < 0.001 | -0.04 |
| CRP | 1.11[0.57-2.39] | 0.99[0.54-2.09] | < 0.001 | -0.052 |
| BMI | 23.22[20.85-26.14] | 23.14[20.87-25.72] | 0.08891 | -0.022 |
| h | 1.58[1.52-1.64] | 1.57[1.52-1.64] | 0.85736 | -0.002 |
| w | 58[50.7-66.2] | 57.7[51-65.5] | 0.33702 | -0.012 |
| WHtR | 0.54[0.49-0.59] | 0.53[0.49-0.58] | < 0.001 | -0.054 |
| BRI | 4.17[3.25-5.25] | 4[3.18-5.02] | < 0.001 | -0.054 |
| CVAI | 100.17[71.06-132.06] | 90.79[64.94-120.28] | < 0.001 | -0.116 |
| WC | 85.1[78-93] | 84.2[77.5-91.4] | < 0.001 | -0.051 |
| CI | 1.29[1.23-1.35] | 1.28[1.22-1.34] | < 0.001 | -0.072 |
| WWI | 11.2[10.64-11.8] | 11.09[10.54-11.68] | < 0.001 | -0.068 |
| HbA1c | 5.1[4.8-5.4] | 5.1[4.9-5.4] | < 0.001 | 0.041 |
| BUN | 15.21[12.69-18.37] | 15.07[12.49-18.12] | 0.04692 | -0.022 |
| UA | 4.38[3.61-5.29] | 4.26[3.55-5.09] | < 0.001 | -0.057 |
| HDL-C | 47.17[38.27-57.6] | 50.26[41.37-60.7] | < 0.001 | 0.114 |
| LDL-C | 2.89[2.34-3.48] | 2.98[2.44-3.58] | < 0.001 | 0.062 |
| eGFR | 98.16[86.17-105.32] | 99.38[89.39-106.27] | < 0.001 | 0.071 |
| CTI | 4.75[4.39-5.22] | 4.68[4.31-5.08] | < 0.001 | -0.091 |
| Sex | female:2465(52.7%); male:2216(47.3%) | female:3850(54.1%); male:3268(45.9%) | 0.13279 | 0.014 |
| Diabetes mellitus | no:3946(84.1%); yes:744(15.9%) | no:6090(85.6%); yes:1028(14.4%) | 0.03665 | 0.019 |
| Residence place | rural:2837(60.5%); urban:1853(39.5%) | rural:4672(65.6%); urban:2446(34.4%) | < 0.001 | 0.052 |
| Smoke | Current:1232(26.3%); Ever:655(14%); Never:2793(59.7%) | Current:2140(30.1%); Ever:578(8.1%); Never:4400(61.8%) | < 0.001 | 0.096 |
| Drink | no:3243(69.3%); yes:1434(30.7%) | no:4701(66%); yes:2417(34%) | < 0.001 | 0.034 |
| Dyslipidemia | no:2571(55.1%); yes:2095(44.9%) | no:4418(62.1%); yes:2700(37.9%) | < 0.001 | 0.069 |
| Hypertension | no:2302(49.2%); yes:2375(50.8%) | no:3287(46.2%); yes:3831(53.8%) | 0.0013 | 0.03 |
| Marita status | Married:4060(86.6%); Other:630(13.4%) | Married:6321(88.8%); Other:797(11.2%) | < 0.001 | 0.033 |
| Education | bove junior high school:553(11.8%); illiterate:2129(45.5%); Junior high school and below:1997(42.7%) | bove junior high school:707(9.9%); illiterate:3383(47.5%); Junior high school and below:3028(42.5%) | 0.00261 | 0.032 |

HbA1c: Glycosylated Hemoglobin, Type A1C; BUN: blood urea nitrogen; UA: uric acid; HDL-C: high density lipoprotein cholesterol; LDL-C: low density lipoprotein cholesterol; eGFR: estimated glomerular filtration rate; CRP: C-reactive protein; TG: triglyceride; FBG: fasting blood glucose; WC:waist circumference.

**Table S19.** Associations between CTI and its related indices(per IQR) and CVD risk in CKM syndrome stage 0-3

| Character | Crude model | | Model 1 | | Model 2 | |
| --- | --- | --- | --- | --- | --- | --- |
|  | 95%CI | P | 95%CI | P | 95%CI | P |
| CTI (per IQR) | 1.28(1.20,1.36) | <0.0001 | 1.24(1.16,1.32) | <0.0001 | 1.18(1.09,1.27) | <0.0001 |
| CTI-BMI(per IQR) | 1.34(1.26,1.41) | <0.0001 | 1.34(1.26,1.42) | <0.0001 | 1.32(1.24,1.42) | <0.0001 |
| CTI-WC(per IQR) | 1.37(1.29,1.46) | <0.0001 | 1.34(1.26,1.43) | <0.0001 | 1.31(1.22,1.42) | <0.0001 |
| CTI-WHtR(per IQR) | 1(1.00,1.00) | <0.0001 | 1(1.00,1.00) | <0.0001 | 1(1.00,1.00) | <0.0001 |
| CTI-WWI(per IQR) | 1.28(1.21,1.37) | <0.0001 | 1.2(1.13,1.28) | <0.0001 | 1.13(1.06,1.22) | <0.001 |
| CTI-BRI(per IQR) | 1.34(1.27,1.42) | <0.0001 | 1.29(1.22,1.38) | <0.0001 | 1.25(1.17,1.34) | <0.0001 |
| CTI-CVAI(per IQR) | 1.42(1.33,1.50) | <0.0001 | 1.34(1.26,1.43) | <0.0001 | 1.34(1.24,1.46) | <0.0001 |
| CTI-CI(per IQR) | 1.29(1.21,1.37) | <0.0001 | 1.22(1.14,1.30) | <0.0001 | 1.16(1.08,1.24) | <0.0001 |

HR Hazard Ratio, CI Confidence Interval

Crude model: unadjusted for covariates;

Model 1: age, sex, marital status, education, eGFR, smoke status, drink status, residence place;

Model 2: age, sex, marital status, education, eGFR, smoke status, drink status, residence place, BUN, UA, LDL-C, HDL-C, HbA1c.

CTI: C-reactive protein-triglyceride-glucose index; CKM: cardiovascular–kidney–metabolic; CVD: cardiovascular disease; WC: waist circumference; WHtR: Waist-to-Height Ratio; WWI: Weight-adjusted waist index; BRI: Body Roundness Index; CVAI: Chinese Visceral Adiposity Index; CI: C-index

**Table S20.** Associations between Cumulative CTI and its related indices(per IQR) and CVD risk in CKM syndrome stage 0-3

| Character | Crude model | | Model 1 | | Model 2 | |
| --- | --- | --- | --- | --- | --- | --- |
|  | 95%CI | P | 95%CI | P | 95%CI | P |
| Cumulative CTI(per IQR) | 1.36(1.23,1.51) | <0.0001 | 1.36(1.22,1.50) | <0.0001 | 1.25(1.10,1.41) | <0.001 |
| Cumulative CTI-BMI(per IQR) | 1.44(1.30,1.59) | <0.0001 | 1.49(1.35,1.66) | <0.0001 | 1.42(1.26,1.60) | <0.0001 |
| Cumulative CTI-WC(per IQR) | 1.45(1.31,1.61) | <0.0001 | 1.46(1.31,1.63) | <0.0001 | 1.38(1.21,1.56) | <0.0001 |
| Cumulative CTI-WHtR(per IQR) | 1.47(1.32,1.63) | <0.0001 | 1.45(1.30,1.61) | <0.0001 | 1.35(1.19,1.53) | <0.0001 |
| Cumulative CTI-WWI(per IQR) | 1.38(1.25,1.52) | <0.0001 | 1.33(1.19,1.47) | <0.0001 | 1.21(1.08,1.37) | 0.002 |
| Cumulative CTI-BRI(per IQR) | 1.45(1.32,1.60) | <0.0001 | 1.43(1.29,1.59) | <0.0001 | 1.34(1.19,1.51) | <0.0001 |
| Cumulative CTI-CVAI(per IQR) | 1.47(1.33,1.62) | <0.0001 | 1.44(1.29,1.59) | <0.0001 | 1.37(1.21,1.56) | <0.0001 |
| Cumulative CTI-CI(per IQR) | 1.38(1.25,1.53) | <0.0001 | 1.34(1.21,1.49) | <0.0001 | 1.23(1.09,1.39) | <0.001 |

HR Hazard Ratio, CI Confidence Interval

Crude model: unadjusted for covariates;

Model 1: age, sex, marital status, education, eGFR, smoke status, drink status, residence place;

Model 2: age, sex, marital status, education, eGFR, smoke status, drink status, residence place, BUN, UA, LDL-C, HDL-C, HbA1c.

CTI: C-reactive protein-triglyceride-glucose index; CKM: cardiovascular–kidney–metabolic; CVD: cardiovascular disease; WC: waist circumference; WHtR: Waist-to-Height Ratio; WWI: Weight-adjusted waist index; BRI: Body Roundness Index; CVAI: Chinese Visceral Adiposity Index; CI: C-index

**Table S21.** Gray’s Test for Cumulative Incidence of CVD and Non-CVD Death by CTI Quartiles

| index | group | χ² | P |
| --- | --- | --- | --- |
| CTI | CVD event | 66.3977 | <0.0001 |
|  | Non−CVD death | 25.7723 | <0.0001 |
| CTI-BMI | CVD event | 89.5736 | <0.0001 |
|  | Non−CVD death | 7.8338 | 0.0496 |
| CTI-WC | CVD event | 91.8000 | <0.0001 |
|  | Non−CVD death | 6.1224 | 0.10508 |
| CTI-WHtR | CVD event | 91.8000 | <0.0001 |
|  | Non−CVD death | 6.1224 | 0.10508 |
| CTI-WWI | CVD event | 73.6416 | <0.0001 |
|  | Non−CVD death | 34.3741 | <0.0001 |
| CTI-BRI | CVD event | 96.3406 | <0.0001 |
|  | Non−CVD death | 4.6164 | 0.2021 |
| CTI-CVAI | CVD event | 119.2599 | <0.0001 |
|  | Non−CVD death | 8.1626 | 0.0428 |
| CTI-CI | CVD event | 78.4069 | <0.0001 |
|  | Non−CVD death | 31.7229 | <0.0001 |

**Table S22.** Association of CTI and its related indices and CVD risk in CKM syndrome stage 0-3 in the Competing Risks Model

| Variable | Event1 | | | | Event2 | | | |
| --- | --- | --- | --- | --- | --- | --- | --- | --- |
|  | subHR | lower95 | upper95 | p | subHR | lower95 | upper95 | p |
| CTI | ref |  |  |  | ref |  |  |  |
| CTI Q2 | 1.2046 | 1.0404 | 1.3947 | 0.013 | 0.9087 | 0.4722 | 1.7486 | 0.770 |
| CTI Q3 | 1.3792 | 1.1903 | 1.5981 | <0.001 | 1.2634 | 0.6835 | 2.3351 | 0.460 |
| CTI Q4 | 1.4529 | 1.2463 | 1.6938 | <0.001 | 2.4511 | 1.3530 | 4.4405 | 0.003 |
| CTI-BMI | ref |  |  |  | ref |  |  |  |
| CTI-BMI Q2 | 1.2461 | 1.0740 | 1.4457 | 0.004 | 0.6627 | 0.3828 | 1.1471 | 0.140 |
| CTI-BMI Q3 | 1.4104 | 1.2150 | 1.637 | <0.001 | 0.7143 | 0.3948 | 1.2921 | 0.270 |
| CTI-BMI Q4 | 1.8312 | 1.5671 | 2.1397 | <0.001 | 1.2444 | 0.6843 | 2.2629 | 0.470 |
| CTI-WC | ref |  |  |  | ref |  |  |  |
| CTI-WC Q2 | 1.2197 | 1.0503 | 1.4164 | 0.009 | 0.8565 | 0.4833 | 1.5179 | 0.600 |
| CTI-WC Q3 | 1.4773 | 1.2734 | 1.7138 | <0.001 | 1.0172 | 0.5573 | 1.8566 | 0.960 |
| CTI-WC Q4 | 1.7001 | 1.4547 | 1.9869 | <0.001 | 1.7295 | 0.9495 | 3.1501 | 0.073 |
| CTI-WHtR | ref |  |  |  | ref |  |  |  |
| CTI-WHtR Q2 | 1.2197 | 1.0503 | 1.4164 | 0.009 | 0.8565 | 0.4833 | 1.5179 | 0.600 |
| CTI-WHtR Q3 | 1.4773 | 1.2734 | 1.7138 | <0.001 | 1.0172 | 0.5573 | 1.8566 | 0.960 |
| CTI-WHtR Q4 | 1.7001 | 1.4547 | 1.9869 | <0.001 | 1.7295 | 0.9495 | 3.1501 | 0.073 |
| CTI-BRI | ref |  |  |  | ref |  |  |  |
| CTI-BRI Q2 | 1.1709 | 1.0081 | 1.3600 | 0.039 | 0.9541 | 0.5520 | 1.6490 | 0.870 |
| CTI-BRI Q3 | 1.3995 | 1.2061 | 1.6238 | <0.001 | 0.8580 | 0.4667 | 1.5776 | 0.620 |
| CTI-BRI Q4 | 1.6699 | 1.4287 | 1.9519 | <0.001 | 1.3880 | 0.7472 | 2.5786 | 0.300 |
| CTI-WWI | ref |  |  |  | ref |  |  |  |
| CTI-WWI Q2 | 1.2165 | 1.0496 | 1.4101 | 0.009 | 0.8078 | 0.3962 | 1.6470 | 0.560 |
| CTI-WWI Q3 | 1.3440 | 1.1591 | 1.5584 | <0.001 | 1.6343 | 0.8609 | 3.1023 | 0.130 |
| CTI-WWI Q4 | 1.4279 | 1.2180 | 1.6739 | <0.001 | 2.6234 | 1.4056 | 4.8965 | 0.003 |
| CTI-CVAI | ref |  |  |  | ref |  |  |  |
| CTI-CVAI Q2 | 1.2548 | 1.0762 | 1.4630 | 0.004 | 0.6415 | 0.3612 | 1.1393 | 0.130 |
| CTI-CVAI Q3 | 1.5376 | 1.3139 | 1.7993 | <0.001 | 0.7160 | 0.3877 | 1.3224 | 0.290 |
| CTI-CVAI Q4 | 1.8491 | 1.5616 | 2.1896 | <0.001 | 0.9294 | 0.5015 | 1.7223 | 0.820 |
| CTI-CI | ref |  |  |  | ref |  |  |  |
| CTI-CI Q2 | 1.1511 | 0.9922 | 1.3353 | 0.063 | 1.2787 | 0.6427 | 2.5439 | 0.480 |
| CTI-CI Q3 | 1.3771 | 1.1897 | 1.5939 | <0.001 | 1.5604 | 0.7991 | 3.0467 | 0.190 |
| CTI-CI Q4 | 1.4429 | 1.2345 | 1.6866 | <0.001 | 2.9655 | 1.5571 | 5.6477 | <0.001 |

Event1: Using complete dataset

Event2: Adjusting the competing risk of deat
